# Pre-bypass ultrafiltration reduces cytokine burden of blood prime in pediatric cardiac surgery

**DOI:** 10.1038/s41598-025-15746-7

**Published:** 2025-08-25

**Authors:** Aylin Poertecene, Svea Kleiner, Leonie Trachte, Sebastian Tiedge, Joerg Optenhoefel, Alexander Horke, Nicole Ruebsamen, Nils Dennhardt, André Karch, Christine Falk, Philipp Beerbaum, Martin Boehne

**Affiliations:** 1 Department of Pediatric Cardiology and Intensive Care Medicine, Hannover Medical School, Carl-Neuberg-Straße 1, 30625 Hannover, Germany; 2 Department of Cardiothoracic, Transplantation, and Vascular Surgery, Hannover Medical School, Hannover, Germany; 3 Institute of Epidemiology and Social Medicine, University Münster, Münster, Germany; 4 Department of Anesthesiology and Intensive Care Medicine, Hannover Medical School, Hannover, Germany; 5 Institute of Transplant Immunology, Hannover Medical School, Hannover, Germany; 6 German Center for Lung Diseases DZL, BREATH, Hannover Medical School, Hannover, Germany; 7 German Center of Infection Research DZIF, TTU-IICH Hannover–Braunschweig site, Hannover, Germany

**Keywords:** Cytokines, Cardiopulmonary bypass, Ultrafiltration, Red blood cell, Priming, Children, Cytokines, Congenital heart defects

## Abstract

Allogeneic red blood cells (RBCs) are commonly used for cardiopulmonary bypass (CPB) circuit priming in congenital heart surgery. While convection-based pre-bypass ultrafiltration (PBUF) corrects acid–base, electrolyte, and metabolite imbalances, its efficacy in removing RBC cytokines/chemokines remains unclear. In a prospective observational study, 22 children (median age: 4.1 months) undergoing congenital heart surgery were enrolled. PBUF of RBC-primed CPB circuits was conducted using bicarbonate-buffered hemofiltration solution. Cytokines/chemokines were quantified in RBC supernatants, CPB priming (before and after PBUF), preoperative patient plasma, and PBUF effluent using Luminex-based multiplex technology. 30 of 50 cytokines were detected in > 50% of RBC supernatants. RBC priming significantly elevated concentrations of 25 cytokines, with 20 further rising after PBUF. At CPB onset, eight mediators (MIF, IL-15, CCL11/Eotaxin, CCL2/MCP-1, VEGF, IL-5, VCAM-1, ICAM-1) exceeded patient plasma concentrations. PBUF filtered cytokines with different efficiencies (0.6–97%). Despite poor filtration or increased concentrations, total mediator load of 42 cytokines decreased significantly (33.3–69.1% of pre-processing levels) after PBUF. In conclusion, PBUF effectively removed multiple cytokines/chemokines released from RBC. Beyond filtration, decrease of total mediator load may be attributed to adsorption to circuit components or rebinding to RBCs. Improved washing techniques may further optimize mediator levels in RBC-primed CPB circuits.

## Introduction

Congenital heart disease (CHD) is the most common congenital malformation^1^. Most children require cardiac surgery with cardiopulmonary bypass (CPB) at an early age^1^. Neonatal and infant congenital heart surgery is further complicated by the significant size mismatch between the CPB system and the patient’s blood volume^2^. Therefore, to prevent excessive hemodilution and severe anemia, whole blood or stored allogeneic red blood cells (RBCs) are often added to the CPB circuit during priming prior to cannulation to maintain a hematocrit above 24% during CBP, as recommended by the Network for the Advancement of Patient Blood Management, Haemostasis and Thrombosis^3^. This practice results in transfusion of stored allogeneic RBCs during initiation of CPB.

RBC transfusion is associated with immunosuppression, infection, electrolyte imbalance, acute kidney injury, and lung injury^4^. These effects are partly related to storage-dependent changes in RBCs, which undergo morphological and functional changes^5^. Recently, packed RBCs have also been shown to be a major reservoir of cytokines, chemokines, and growth factors, suggesting a potential role in inflammatory processes^5,6^. In addition, stored RBCs develop unphysiologic acid–base, electrolyte, and metabolite values^7^. To optimize unprocessed RBCs for CPB circuit priming, the American Society of ExtraCorporeal Technology recommends pre-bypass ultrafiltration (PBUF)^2^. At our institution, using a bicarbonate-buffered solution for PBUF, improved electrolyte, lactate, and acid–base levels of the priming solution^8^. However, the ability of PBUF to remove pro-inflammatory mediators from packed RBCs used for CPB priming has not been evaluated.

Therefore, we conducted a single-center, prospective observational study to assess the inflammatory mediator burden of allogeneic RBC-primed CPB circuits in neonatal and infant congenital heart surgery, as well as the efficacy of PBUF in reducing cytokine/chemokine levels delivered to the patient.

## Results

### Study population

22 children with various congenital heart defects with a median (range) age of 4.1 months (10 days to 34 months), a median weight of 5.0 (3.1.−10.5) kg and Risk Adjustment for Congenital Heart Surgery score of 3 (2–6) were included in the study (Supplementary Table S1). Two children were treated in the intensive care unit prior to surgery.

**Fig. 1:**
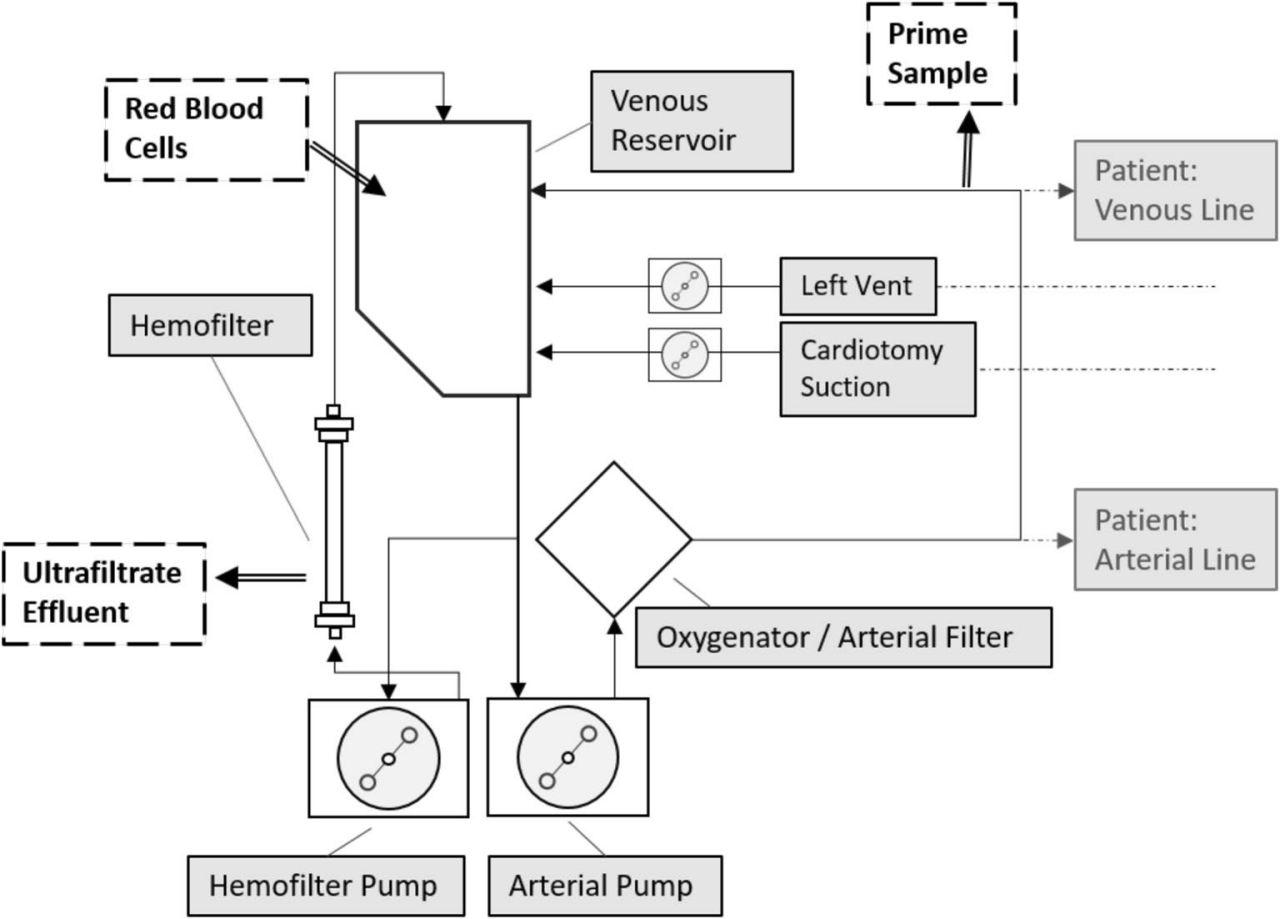
Standardized cardiopulmonary bypass (CPB) circuit setup for all 22 patients. Prime samples were collected at the venous line during the priming process. Pre-bypass ultrafiltration (PBUF) effluent samples were directly taken from the hemofilter outlet at the end of the priming process.

PBUF was conducted with a median flow rate (range) of 0.125 L/min (0.05–0.21) during each surgery at the perfusionists’ discretion until pH, metabolic parameters and electrolytes of the CPB circuit prime reached normal levels (Fig. 1). This required a median duration of 10 min (4–35) and a median volume of 400 mL (250–770) of hemofiltration solution.

### Mediator burden in RBC supernatants

**Fig. 2:**
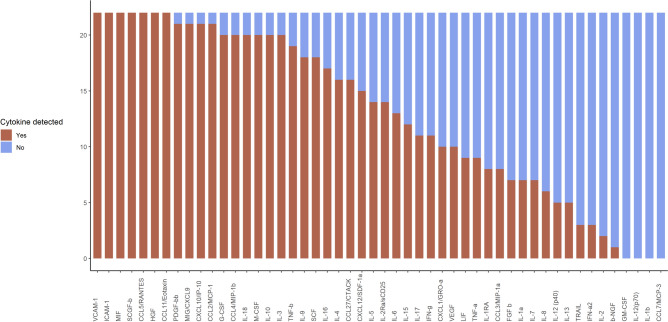
Cytokines in packed red blood cells (RBCs). Proportion of mediator-positive units of red blood cell (RBC) concentrates from n = 22 samples used for pediatric cardiopulmonary bypass circuit priming for 50 measured cytokines.

Quantification of cytokines was performed on 22 RBC units obtained immediately before their application to the CPB prime (Table 1). Of the 50 cytokines assayed, 46 were detected in at least one RBC supernatant showing a high inter-sample variability, with 30 of the 50 mediators detected in at least 50% of the RBC supernatants. Notably, 16 of the 50 mediators were present in more than 90% of RBC supernatant samples (Fig. 2). Only 6 mediators, namely Interleukin (IL)−1β, IL-12p70, IL-2, Monocyte chemotactic protein 3 (CCL7/MCP-3), Nerve growth factor β (β-NGF) and Granulocyte–macrophage colony-stimulating factor (GM-CSF), were detectable in 10% or less of RBC supernatants. For details on RBC storage, please refer to the Supplementary Information.

**Table 1:** Concentrations (pg/mL) of the 50 cytokines during prime preparation.

	Cytokine	RBC		Priming solution before PBUF		Priming solution after PBUF		Patient baseline	
		Median (IQR)	(Out of 22)	Median (IQR)	(Out of 22)	Median (IQR)	(Out of 22)	Median (IQR)	(Out of 16)
		[pg/ml]		[pg/ml]		[pg/ml]		[pg/ml]	
pro-inflammatory	MIF	3410.89 (3011.62)	(22)	1818.06^y^ (1349.98)	(22)	1902.82 (1517.94)	(22)	82.43^zzz^ (20.07)	(16)
	IL-15	225 (111.66)	(12)	322.23^yy^ (82.96)	(17)	337.26 (106.91)	(18)	221.33^zzz^ (69.17)	(4)
	IL-9	10.67 (22.19)	(18)	39.05^yy^ (34.79)	(22)	46.60* (41.96)	(22)	85.14^zz^ (34.61)	(16)
	TNF-β	9.57 (19.77)	(19)	35.35^yy^ (27.40)	(22)	48.44** (42.66)	(22)	82.21^z^ (39.49)	(16)
	IL-18	6.71 (5.56)	(20)	2.95^y^ (2.52)	(18)	5.07 (3.18)	(18)	11.08^zz^ (9.41)	(16)
	TRAIL	4.44 (0.96)	(3)	4.76^yy^ (5.89)	(13)	7.52 (8.49)	(12)	20.55^zzz^ (12.50)	(16)
	IL-1α	3.19 (2.44)	(7)	2.38 (2.44)	(13)	3.99** (2.39)	(16)	18.4^zzz^ (18.03)	(16)
	TNF-α	1.56 (1.40)	(9)	3.46 (3.51)	(13)	3.16 (2.39)	(13)	12.01^zzz^ (6.42)	(16)
	IL-17	0.73 (0.70)	(11)	1.40^yyy^ (1.85)	(20)	2.04* (2.45)	(19)	4.16^zzz^ (3.19)	(16)
	IFN-γ	0.16 (0.43)	(11)	0.89^yyy^ (0.59)	(18)	1.30* (1.14)	(21)	2.58^zz^ (1.81)	(16)
	IL-1β	n.a.	(0)	0.03 (0)	(1)	0.37 (0.04)	(2)	0.58^zzz^ (0.58)	(11)
	IL-12(p70)	n.a.	(0)	n.a.	(0)	n.a.	(0)	1.77^zzz^ (1.12)	(10)
	IL-2Rα	3.85 (1.43)	(14)	33.13^yyy^ (18.45)	(21)	39.9** (23.19)	(22)	36.02 (20.65)	(16)
anti-inflammatory	IL-1RA	162.19 (120.12)	(8)	84.48 (53.85)	(6)	111.4 (81.52)	(11)	255.67^zzz^ (180.23)	(16)
	IL-13	1.59 (1.20)	(5)	0.39 (1.55)	(11)	1.94** (1.49)	(14)	5.21^zzz^ (2.13)	(16)
	IL-10	1.20 (0.65)	(20)	1.70 (1.33)	(20)	1.7 (1.52)	(21)	5.52^zzz^ (3.17)	(16)
	IL-4	1.19 (0.92)	(16)	1.93^yy^ (1.92)	(21)	2.2** (2.45)	(22)	3.36 (1.00)	(16)
multifunctional	IL-12(p40)	5.74 (5.87)	(5)	27.38 (53.06)	(9)	11.61 (13.09)	(7)	62.45^zzz^ (44.26)	(15)
	IFN-α2	3.27 (1.83)	(3)	4.33 (2.92)	(8)	4.41 (2.12)	(10)	15.10^zzz^ (4.67)	(16)
	LIF	2.66 (0)	(9)	8.04^yy^ (8.53)	(14)	16.62 (12.04)	(14)	45.38^zzz^ (33.88)	(16)
	IL-6	0.68 (0.53)	(13)	1.25 (0.67)	(15)	1.25 (0.56)	(17)	0.83 (1.03)	(9)
	IL-2	0.20 (0.18)	(2)	0.35 (0.66)	(6)	0.68 (0.28)	(7)	3.34^zzz^ (2.23)	(13)
chemokines	GRO-α/CXCL1	232.24 (95.26)	(10)	279.50^yy^ (73.42)	(19)	298.23 (63.36)	(20)	306.08 (89.70)	(14)
	RANTES/CCL5	62.41 (62.44)	(22)	188.64^yyy^ (264.68)	(22)	224.01 (256.78)	(22)	317.06 (136.84)	(16)
	Eotaxin/CCL11	15.65 (18.32)	(22)	68.32^yyy^ (56.79)	(22)	92.49** (66.75)	(22)	7.28^zzz^ (4.54)	(16)
	MIG/CXCL9	14.51 (6.29)	(21)	12.46 (6.54)	(20)	10.02 (6.67)	(21)	57.79^zzz^ (89.75)	(16)
	SDF-1α	9.60 (8.59)	(15)	16.79^yy^ (11.34)	(19)	20.00 (12.71)	(20)	104.39^zzz^ (49.59)	(16)
	MIP-1β/CCL4	9.52 (10.43)	(20)	19.76^yy^ (12.66)	(22)	24.01* (13.35)	(22)	39.20^zz^ (10.41)	(16)
	IP-10/CXCL10	8.39 (7.10)	(21)	10.48 (7.23)	(21)	11.57 (9.14)	(22)	71.99^zzz^ (60.15)	(16)
	MCP-1/CCL2	4.32 (3.68)	(21)	16.06^yyy^ (11.65)	(22)	22.38*** (15.95)	(22)	6.38^zzz^ (2.76)	(16)
	IL-16	3.43 (6.12)	(17)	2.32 (4.47)	(20)	4.44*** (2.85)	(17)	20.96^zzz^ (10.09)	(16)
	CTACK/CCL27	0.83 (1.82)	(16)	0.35 (1.32)	(16)	0.83 (0.65)	(19)	102.68^zzz^ (50.92)	(16)
	IL-8/CXCL8	0.71 (0.87)	(6)	1.14^yy^ (1.06)	(13)	1.86* (0.93)	(14)	2.43^z^ (2.99)	(15)
	MIP-1α CCL3	0.24 (0)	(8)	0.40 (0)	(1)	0.24 (0)	(1)	1.42^zzz^ (0.43)	(16)
	MCP-3/CCL7	n.a.	(0)	n.a.	(0)	0.37 (0)	(2)	2.65^zzz^ (1.83)	(15)
growth factors	SCGFβ	1549.97 (1077.02)	(22)	1464.12 (742.67)	(22)	1464.06 (737.51)	(22)	12,755.30^zzz^ (5290.83)	(16)
	VEGF	51.89 (42.26)	(10)	131.23^yy^ (70.41)	(17)	114.34 (91.74)	(18)	98.58^zzz^ (0)	(1)
	IL-5	51.14 (43.28)	(14)	108.57^y^ (74.09)	(18)	110.43 (56.66)	(18)	184.09^zzz^ (0)	(1)
	PDGF-bb	19.96 (7.41)	(21)	23.66 (7.39)	(21)	27.35 (16.62)	(21)	91.30^zzz^ (35.46)	(16)
	HGF	17.54 (16.00)	(22)	10.10^yy^ (9.14)	(19)	12.01 (12.47)	(20)	70.24^zzz^ (25.29)	(16)
	G-CSF	10.30 (7.08)	(20)	21.28^yy^ (15.89)	(22)	29.54*** (22.17)	(22)	55.90^zzz^ (29.60)	(16)
	FGF-β	4.86 (3.40)	(7)	1.46 (1.11)	(8)	4.86* (5.54)	(12)	19.43^zzz^ (6.31)	(16)
	SCF	3.32 (3.23)	(18)	21.15^yyy^ (15.20)	(21)	28.35* (25.07)	(22)	22.47 (8.27)	(16)
	IL-7	2.39 (3.33)	(7)	4.75^y^ (3.67)	(14)	8.42** (6.26)	(17)	19.63^zzz^ (9.23)	(16)
	M-CSF	2.05 (1.04)	(20)	10.92^yyy^ (10.17)	(22)	13.12* (11.37)	(22)	9.15 (6.04)	(16)
	IL-3	0.78 (0.70)	(20)	1.51^yy^ (0.67)	(21)	1.41 (0.75)	(21)	1.13 (0.77)	(13)
	β-NGF	0.12 (0)	(1)	1.36^yy^ (0.75)	(8)	1.57* (1.54)	(14)	1.28 (0.86)	(6)
	GM-CSF	n.a.	(0)	2.14 (1.14)	(3)	2.89 (1.70)	(6)	3.43^zz^ (2.53)	(12)
	VCAM-1	35,101.32 (14,283.12)	(22)	11,163.93^yyy^ (9155.48)	(22)	13,614.31* (8532.63)	(22)	7852.70^zz^ (4378.05)	(16)
	ICAM-1	13,915.28 (7498.80)	(22)	44,398.93^yyy^ (23,698.39)	(22)	52,886.05* (27,745.60)	(22)	4002.53^zzz^ (1838.18)	(16)

Red blood cell (RBC): sample of red blood cell concentrate; Priming solution before pre-bypass ultrafiltration (PBUF): sample from the CPB circuit after complete priming with RBC and crystalloid solutions before PBUF; Priming solution after PBUF: sample from the CPB circuit following PBUF. Patient baseline: blood sample after anesthesia induction and central venous line placement. The table was sorted according to functional cytokine groups, and within these groups, according to cytokine concentration levels in RBCs.

Median and interquartile range (IQR) and number of cytokine-positive samples in brackets are shown for each cytokine. Concentration of cytokine in RBC versus priming solution before PBUF, Wilcoxon Signed-Rank Test, y p < 0.05; yy p < 0.01; yyy p < 0.001; Priming solution before PBUF versus priming solution after PBUF, Wilcoxon Signed-Rank Test, * p < 0.05; ** p < 0.01; *** p < 0.001; Priming solution after PBUF versus Patient baseline, Wilcoxon Signed-Rank Test, z p < 0.05; zz p < 0.01; zzz p < 0.001.

CTACK, Cutaneous TCell attracting chemokine (CCL27); FGF-β, Basic fibroblast growth factor; G-CSF, Granulocyte--colony stimulating factor; GM-CSF, Granulocyte–macrophage colony-stimulating factor; GRO-α, Growth-regulated alpha protein (CXCL1); HGF, Hepatocyte growth factor; ICAM, Intracellular adhesion molecule; IFN-γ, Interferon-gamma; IL, Interleukin; IL-1RA, Interleukin receptor antagonist 1; IL-2Rα, soluble IL-2 receptor alpha, IP-10, Interferon-gamma-inducible protein 10 (CXCL10); LIF, Leukemia inhibitory factor; MCP, Monocyte chemotactic protein; M-CSF, Macrophage colony-stimulating factor; MIF, Macrophage migration inhibitory factor; MIG, Monokine induced by interferon gamma (CXCL9); MIP, Macrophage inflammatory protein; n.a., not applicable; NGF, Nerve growth factor; PDGF-bb, Platelet-derived growth factor-BB; RANTES, Regulated upon activation normal T cell expressed and presumably secreted (CCL5); SCF, Stem cell factor; SCGF, Stem cell growth factor; SDF-1α, Stromal cell-derived factor 1α (CXCL12); TNF, Tumor necrosis factor; TRAIL, Tumor necrosis factor-related apoptosis-inducing ligand; VCAM, Vascular cell adhesion molecule; VEGF, Vascular endothelial growth factor.

### Cytokine concentration in RBC-free priming

To exclude relevant cytokine contamination from crystalloid priming solution ingredients, additional samples were collected from the asanguine, crystalloid priming solution before RBC were added, showing low levels of Tumor necrosis factor-related apoptosis-inducing ligand (TRAIL), soluble IL-2 receptor alpha (IL-2Rα), Regulated upon activation normal T cell expressed and presumably secreted (CCL5/RANTES), Stem cell growth factor β (SCGFβ), Stem cell factor (SCF) and Macrophage colony-stimulating factor (M-CSF); all other cytokines were not detected at quantifiable levels (Supplementary Table S2).

### Mediator concentration during priming and PBUF

**Fig. 3:**
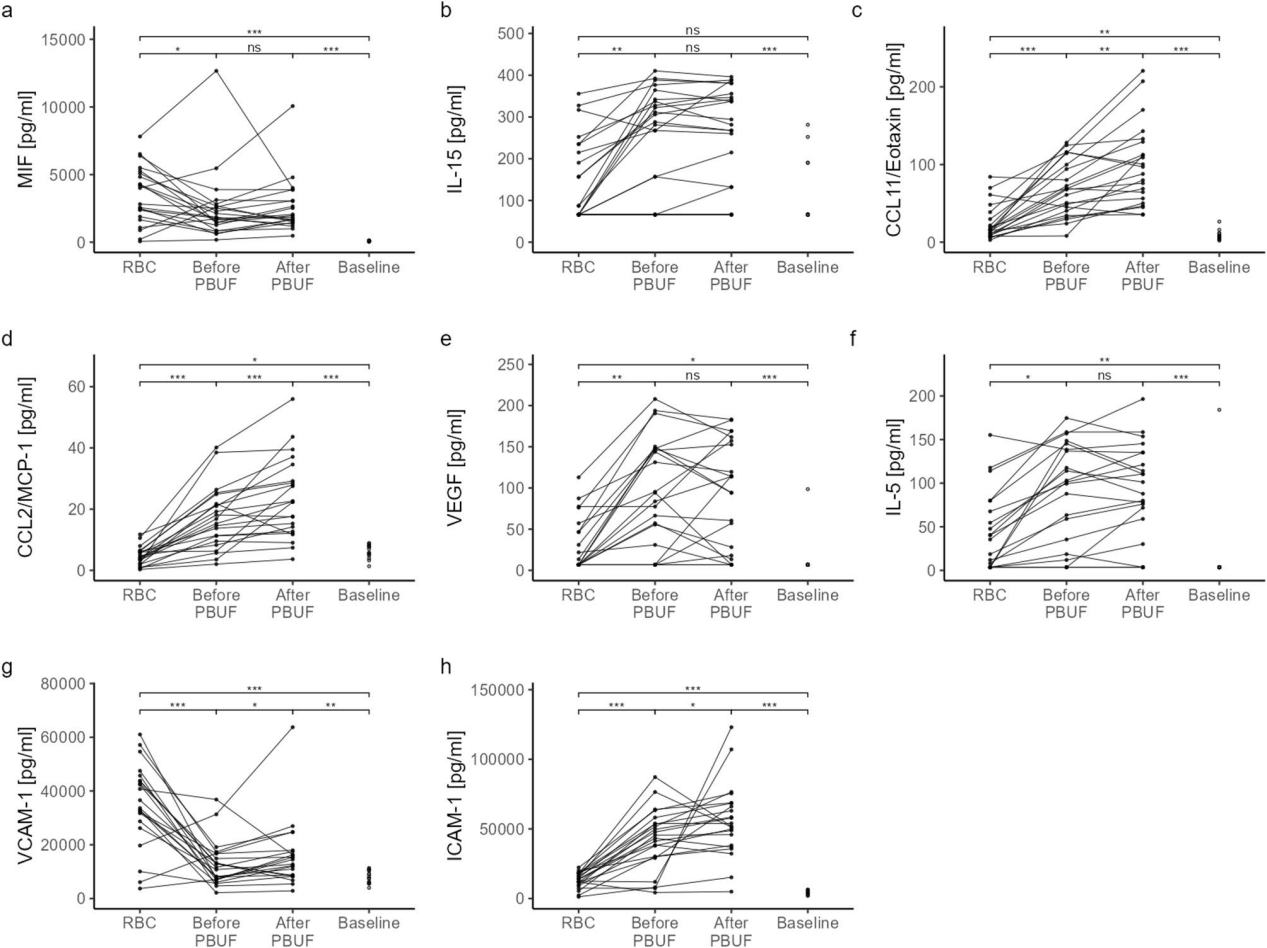
Longitudinal course of cytokine concentrations during priming. **a**-**h**) Longitudinal course of 8 cytokines out of 50 during prime preparation. Figures show mediator concentrations in red blood cell (RBC) supernatants and prime samples before pre-bypass ultrafiltration (PBUF) and after PBUF. After PBUF, the concentrations of eight cytokines (MIF, IL-15, CCL11/Eotaxin, CCL2/MCP-1, VEGF, IL-5, VCAM-1 and ICAM-1) exceeded the median patient baseline concentration, while the remaining cytokines were at or below patient baseline. *p < 0.05, **p < 0.01, ***p < 0.001; see Supplementary Figure S2 for figures of the remaining 42 cytokines. ICAM, Intracellular adhesion molecule; IL, Interleukin; MCP, Monocyte chemotactic protein, MIF, Macrophage migration inhibitory factor; VCAM, Vascular cell adhesion molecule; VEGF, Vascular endothelial growth factor.

After priming of the CPB circuit, circulation and homogeneous mixing of all prime components, all cytokines identified in RBC supernatants remained detectable. Most mediators were present at either comparable or higher concentrations than in RBC supernatants (Table 1). Of the 50 mediators analyzed, the concentrations of 25 (IL-15, IL-9, Tumor necrosis factor beta [TNF-β], TRAIL, IL-17, Interferon-gamma [IFN-γ], IL-2Rα, IL-4, Leukemia inhibitory factor [LIF], Growth-regulated alpha protein [CXCL1/GRO-α], CCL5/RANTES, CCL11/Eotaxin, Stromal cell-derived factor 1 alpha [CXCL12/SDF-1α], Macrophage inflammatory protein 1β [CCL4/MIP-1β], Monocyte chemotactic protein 1 [CCL2/MCP-1], CXCL8/IL-8, Vascular Endothelial Growth Factor [VEGF], IL-5, Granulocyte-Colony Stimulating Factor [G-CSF], SCF, IL-7, Macrophage colony-stimulating factor [M-CSF], IL-3, β-NGF, Intracellular adhesion molecule 1 [ICAM-1]) increased significantly, while 4 cytokines (Macrophage migration inhibitory factor [MIF], IL-18, Hepatocyte growth factor [HGF] and soluble Vascular cell adhesion molecule 1 [VCAM-1]) decreased significantly in concentration. For the remaining cytokines, no change in concentration was observed (Fig. 3 and Table 1, Supplementary Figure S2). Irrespective of the changes in concentration, many of the mediators were detected in a larger number of priming samples than in the RBC samples.

After complete assembly, the priming solution was circulated in the CPB circuit and PBUF was performed. After PBUF, 20 out of 50 cytokines further increased significantly in concentration (IL-9, TNF-β, IL-1α, IL-17, IFN-γ, IL-2Rα, IL-13, IL-4, CCL11/Eotaxin, CCL4/MIP-1β, CCL2/MCP-1, CXCL8/IL-8, G-CSF, basic fibroblast growth factor [FGF-β], SCF, IL-7, M-CSF, β-NGF, VCAM-1, ICAM-1), while the others remained stable, none decreased (Table 1 and Fig. 3, Supplementary Figure S2). Some of the cytokines (IL-1α, IFN-γ, IL-1 RA, IL-13, CCL27/CTACK, FGF-β, IL-7, β-NGF) were also detected in a relevant higher number of samples than before PBUF. Only IL-12p70 was undetectable in both, pre- and post-PBUF samples (Table 1).

### Priming vs. patient mediator concentration

To establish a threshold for the potential clinical relevance of mediator burden in RBC priming, we compared the cytokine levels detected in the priming after PBUF with the median preoperative plasma levels of the patients in our study cohort for each of the cytokines. After PBUF, the concentration of 8 out of 50 cytokines (MIF, IL-15, CCL11/Eotaxin, CCL2/MCP-1, VEGF, IL-5, VCAM-1, and ICAM-1) in the CPB circuit significantly exceeded the median preoperative baseline levels of the patients (Table 1, Fig. 3, Supplementary Figure S2). Levels of all other mediators showed a non-significant trend to be elevated above the patient baseline (IL-3, IL-4, M-CSF [p-value < 0.1]), ranged below the median patient baseline, or were undetectable (Table 1).

### Mediator removal by PBUF

**Fig. 4:**
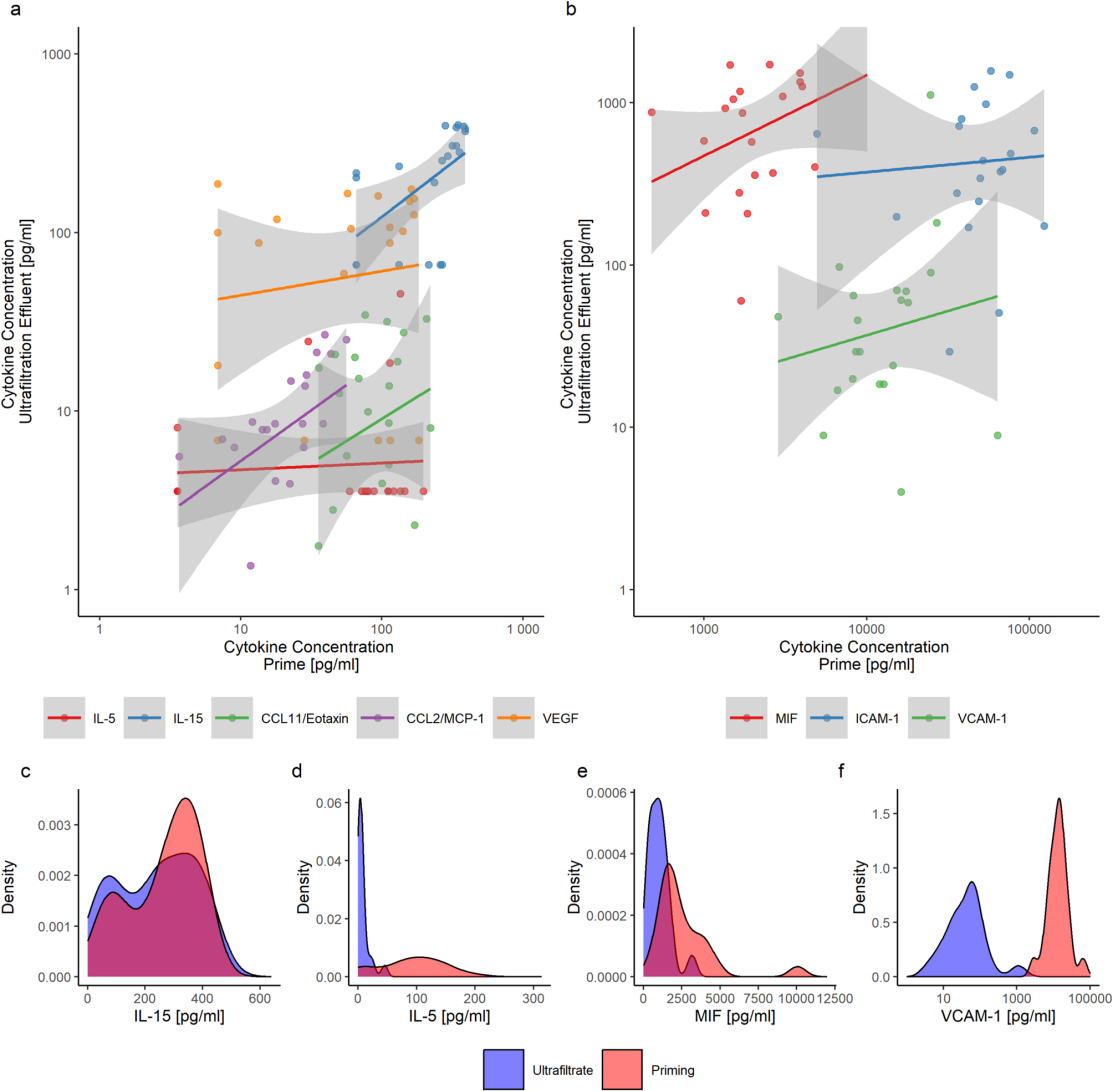
Cytokine concentrations in pre-bypass ultrafiltration effluent. **a**-**b**) Figure illustrates the spot concentrations of cytokines in the pre-bypass ultrafiltration (PBUF) effluent during the terminal phase of PBUF in relation to the concomitant mediator concentrations of CPB priming in **a**) low concentration range and **b**) high concentration range. The selection of these eight cytokines presented was based on their levels in the prime after PBUF, which exceeded the patient’s baseline. **c**-**f**) The figure depicts the density plots of the overall distribution of cytokine concentrations in CPB priming (red) and PBUF effluent (blue) for four exemplary cytokines. The height of the curve at a given point along the x-axis indicates the relative likelihood of the concentration of IL-15, IL-5, MIF and VCAM-1 in the respective sample type. Strong overlap indicates convergence of cytokine concentrations in the PBUF effluent and therefore more efficient cytokine removal.

The majority of the 50 cytokines analyzed were also detectable in the PBUF effluent at the end of PBUF (Supplementary Table S3). Among the 8 cytokines whose concentrations in the priming solution after PBUF exceeded baseline serum levels in patients, 7 showed a positive correlation between their spot concentrations in the PBUF effluent and their respective levels in the CPB prime. The slope of the linear regression line varied for each cytokine, reflecting differences in filtration properties (Fig. 4, Supplementary Figure S3).

The removal effect, calculated as ratio of respective cytokine concentrations in the priming and PBUF effluent, ranged from 0.67 ± 1.03% for VCAM-1 to 97.73 ± 83.92% for VEGF (Table 2, Supplementary Table S3). However, there was variability in the removal effect, with better filtration of molecules such as VEGF (43 kDa) or IL-15 (18 k Da) and restricted filtration of molecules such as M-CSF (107 kDa), ICAM-1 (58 kDa in monomeric form), and VCAM-1 (81–110 kDa).

**Table 2:** Filtration of selected cytokines.

Cytokine	Priming solution after PBUF		PBUF effluent		PBUF removal effect
	Median (IQR)	(Out of 22)	Median (IQR)	(Out of 19)	(SD)
	[pg/ml]		[pg/ml]		
MIF	1902.82 (1517.94)	(22)	923.20 (811.77)	(19)	50.41% (42.50%)
IL-15	337.26 (106.91)	(18)	305.89 (136.21)	(13)	85.73% (52.65%)
CCL11/Eotaxin	92.49 (66.75)	(22)	13.82 (15.06)	(19)	18.69% (15.06%)
CCL2/MCP-1	22.38 (15.95)	(22)	8.50 (9.08)	(19)	54.66% (31.58%)
VEGF	110.43 (56.66)	(18)	122.28 (57.94)	(14)	97.73% (83.92%)
IL-5	27.35 (16.62)	(21)	18.59 (16.50)	(5)	8.97% (22.11%)
VCAM-1	2.89 (1.70)	(6)	53.41 (48.79)	(18)	0.67% (1.03%)
ICAM-1	13,614.31 (8532.63)	(22)	486.5 (576.71)	(19)	2.19% (3.22%)

Comparison of the concentration (pg/mL) of 8 out of 50 cytokines that were detected at or above the median preoperative baseline values in our patient cohort. Concentrations are shown for the priming solution after pre-bypass ultrafiltration (PBUF) and in the PBUF effluent. Different removal effects were observed for individual cytokines. A comprehensive list of all mediator concentrations and their removal effect by PBUF is provided in Supplementary Table S3.

CCL, Chemokine C–C motif ligand; ICAM, Intracellular adhesion molecule; IL, Interleukin; IQR, Interquartile range; MCP, Monocyte chemotactic protein; MIF; Macrophage migration inhibitory factor; PBUF, Pre-bypass ultrafiltration; SD, standard deviation; VCAM, Vascular cell adhesion molecule, VEGF, Vascular endothelial growth factor.

### Absolute mediator content during priming and PBUF

To further estimate the total mediator load, the mediator concentration was multiplied by i) the volume of applied RBCs and ii) the respective filling volume of the CPB circuit at specific stages of the priming process, accounting for the particular volume used for PBUF.

After priming the CPB circuit, the total mediator load for 48 cytokines increased significantly compared to the mediator load from the applied packed RBC volume (Supplementary Figure S4 and Table S4). CCL7/MCP-3 and IL-12(p70), which were below the lower detection limit in RBCs, were not detectable.

After further processing of the prime comprising PBUF, the load of 42 out of 50 mediators decreased significantly (Supplementary Figure S4 and Table S4), whereas the contents of 5 mediators did not decrease significantly (IL-2, β-NGF, GM-CSF) or remained stable (IL-13, FGF-β). These 5 mediators were consistently detected at low concentrations in all samples (Table 1). Only IL-1β and CCL7/MCP-3 levels increased significantly, but these were detected in only 1 or 2 samples above the detection limit. IL-12(p70) was undetectable.

The final mediator load ranged from 33.3% (CXCL9/MIG) to 69.1% (IL-16) of pre-PBUF baseline levels. Notably, mediators that exceeded the median preoperative baseline concentrations in our patient cohort, as well as those identified to be poorly filtered (Table 2), were also significantly reduced.

## Discussion

We conducted a single-center observational study to assess the inflammatory mediator burden of allogeneic RBC-primed CPB circuits during neonatal and infant congenital heart surgery, as well as the efficacy of pre-bypass ultrafiltration (PBUF) in reducing cytokine and chemokine levels. To the best of our knowledge, this is the first comprehensive analysis of a broad range of pro- and anti-inflammatory factors, chemokines, growth factors and endothelial markers during the priming process. Our findings confirm that packed RBC, once thought to be immunologically inert, are sources of a wide variety of cytokines. After RBC priming of the CPB circuit, circulation and homogeneous mixing of all components, RBCs continue to release mediators, resulting in higher cytokine concentrations and loads in CPB prime compared to packed RBC supernatants. Subsequent PBUF was highly effective and successfully removed a significant portion of mediators through the effluent. Notably, our study is the first to demonstrate that the entire priming process further reduced the overall inflammatory mediator load.

First, most of the cytokines we found in packed RBC were detectable at low levels in the supernatants of RBC units, with a large variability most likely due to donor specifications. This observation is consistent with recent studies, including those by Karsten et al., which highlighted RBCs as a dynamic cytokine reservoir^5,9^. Their investigation identified 46 mediators in RBC lysates and 46 cytokines in RBC-conditioned media^5,9^. Our analysis supplements their findings, detecting 46 out of 50 mediators in RBC units used for CPB priming, with 30 present in more than half of the stored RBC unit supernatants. However, we observed slight differences in the detectable cytokines. Four mediators (IL-1β, IL-12 (p70), CCL7/MCP-3, and GM-CSF) previously reported in RBC-conditioned media were undetectable in our RBC supernatant samples^5,9^, while three cytokines (CCL7/MCP-3, β-NGF, and SCGF-ß) were not previously described^5,9^. These discrepancies may be due to high inter-individual and donor-dependent cytokine variability in RBC units and limited sample sizes in both studies. Additionally, while Karsten et al. used freshly isolated RBCs for their experiments, we investigated packed RBC after routine handling and storage.

Second, while crystalloid priming revealed only trace amounts of a few cytokines before the addition of RBC, the RBCs continue to release mediators after administration into the CPB circuit. This resulted in significantly higher cytokine levels in CPB prime compared to initial RBC supernatants, suggesting an ongoing release mechanism, likely amplified by mechanical stimulation from CPB circuit components^10^. It is reasonable to assume that shear stress and surface contact with circuit components induce some degree of hemolysis, resulting in the release of intracellular mediators from RBC^5,10,11^. However, the literature indicates that a fraction of cytokines, such as CXCL8/IL-8, exists in a bound form within the RBC cytoplasm and might therefore not be detectable in our assays after hemolysis^12^. Additional active release of monomeric cytokines by intact RBCs is likely to cause a measurable increase in mediator levels throughout the priming preparation^5^. To that effect, versatile binding and adaptive release of mediators on RBCs through the Duffy antigen receptor for chemokines (DARC) and the erythrocyte glycocalyx have been previously described^5^. To date, two major triggers for CCL2/MCP-1 release from DARC have been identified: the exposure to unfractionated heparin and the coagulation process^13^. Both conditions are frequently encountered when donor blood is used for CPB priming.

Third, PBUF of the CPB prime was effective, successfully removing a significant portion of the mediator load, as indicated by cytokines detection in the PBUF effluent. The concomitant increase in the concentration of 20 out of 50 inflammatory mediators after PBUF is likely due to several factors. As a convection-based process, PBUF cannot achieve an absolute reduction in mediator concentrations^14^. The effectiveness of filtration is further constrained by the filter membrane properties and the specific characteristics of each cytokine^15,16^. According to the manufacturer^17^, a comparable larger hemofilter with an identical polyarylsulfone membrane used in our study exhibits a sieving coefficient of 0.6 for 10 kDa molecules. Since most chemokines have a molecular mass of approximately 15 kDa^18^, they are expected to be effectively filtered, whereas larger molecules are known to be less removed^15^. Notably, some mediators exhibited unexpected filtration properties relative to their molecular weight. For instance, SCF (30 kDa) was largely undetectable in PBUF effluent, while IL-5 (15 kDa) appeared only in a subset of effluent samples (5 out of 21). This suggests that probably additional factors such as hydrophobicity or molecular structure may influence cytokine removal by PBUF^15,16^. To the best of our knowledge, no investigation has addressed how structural heterogeneity of individual mediators affects their filtration through hemofiltration membranes. This issue is particularly relevant because, despite being primarily monomeric, structural analyses have demonstrated that many soluble mediators can form dimers or higher-order oligomers and interact transiently with plasma proteins under physiological and pathophysiological conditions. These dynamic structural changes can substantially alter key physicochemical properties, such as hydrodynamic radius, surface charge, and overall conformation^19,20^. These properties may determine membrane permeability and, consequently, clearance efficiency during hemofiltration.

Removal efficiency by PBUF varied from 0.6% to 97% across different cytokines, which is consistent with findings from other studies, such as Bierer et al., who documented extensive removal of 20 out of 39 mediators via ultrafiltration^16^. The low mediator concentrations in CPB prime preclude drawing generalized conclusions about the removal capacity for some of the cytokines that were undetectable in PBUF effluent. However, a difference was found for CCL5/RANTES, which was well filtered in our system, but was not removed in the study by Bierer et al^16^. This difference may be attributed to the use of different filtration membranes: polysulfone in their study versus polyarylsulfone in ours. Both materials likely have different filtration characteristics for specific cytokines, as previously published for comparison of polyamide and polysulfone membranes^21^.

Overall, we observed a significant reduction in the total load of 42 different mediators during the entire priming processing. The final mediator load ranging from 33.3% (CXCL9/MIG) to 69.1% (IL-16) of pre-processing baseline levels. This reduction is primarily attributed to the PBUF described above; however, even mediators considered to be poorly filtered showed a significant decrease in load, suggesting additional mechanisms, not specifically addressed in our study, to be involved. Beyond filtration, some polymers used in hemofiltration have been reported to adsorb various inflammatory cytokines^22^, thereby lowering inflammatory mediator levels. Cytokine adsorption to CPB components, especially to the inner heparin-coated surfaces^23^, or binding to circulating albumin^24^ may also contribute. Furthermore, re-binding of mediators to RBCs through DARC or glycocalyx could also be a relevant mechanism^5^.

At our institution, sanguineous CPB prime is used when indicated to maintain recommended intraoperative hematocrit levels in neonates and infants^3^. To assess the potential clinical relevance of cytokine levels detected in CPB prime after PBUF, we used the median baseline concentration of patients measured in plasma samples taken before sternotomy as a threshold. At the time of CPB cannulation, the concentration of 8 out of 50 cytokines (MIF, IL-15, CCL11/Eotaxin, CCL2/MCP-1, VEGF, IL-5, VCAM-1, ICAM-1) in the prime exceeded those in patient plasma. Among these, MIF and CCL2/MCP-1 constitutes pro-inflammatory chemotactic cytokines that affect monocyte and macrophage migration and differentiation^25,26^. CCL2/MCP-1 is involved in multiple inflammatory diseases^27^, while MIF is a pleiotropic protein with multiple biological functions playing a critical role in a variety of infectious and autoimmune diseases^28^, as well as in kidney injury^29^. Erythrocytes have been shown to be the largest reservoir of MIF in the blood^5,9^. We also detected T helper type 2-associated eosinophil chemoattractants like IL-5^30^ and CCL11/Eotaxin^31^ as well as pleiotropic pro-inflammatory mediators such as IL-15^32^. The cell adhesion molecules ICAM-1 and VCAM-1 belong to the immunoglobulin superfamily and primarily play roles in leucocyte migration, endothelial adherence and lymphocyte activation^33^. Upon inflammatory activation of endothelial cells, soluble forms are released into the bloodstream, serving as a marker of endothelial activation after cardiopulmonary bypass^34^.

It is challenging to assess the clinical impact of cytokine exposure from RBC-primed CPB within the complex inflammatory environment of neonatal and infant congenital heart surgery. However, recent clinical studies of these inflammatory mediators in pediatric cardiac surgery, as well as existing data from adults and other clinical contexts, suggest that these mediators likely play an important role. In adult cohorts, promoter polymorphisms in the MIF and VEGF genes have been associated with an increased incidence of acute kidney injury and mortality following cardiac surgery^35,36^. Higher postoperative MIF levels correlated with prolonged need for mechanical ventilation and increased inotropic support in pediatric patients^28^ as well as with pulmonary dysfunction and higher doses of vasopressors in adults^37^. In neonates, preoperative VEGF levels have been linked to the severity of postoperative capillary leak syndrome^38^. Likewise, elevated baseline VCAM-1 expression has been related to prolonged postoperative hospital stays^39^. Although specific data on IL-15 in the context of cardiac surgery is limited, IL-15 plays a central role in activating natural killer (NK) cells and T lymphocytes. Elevated IL-15 levels have been associated with more severe systemic inflammatory response syndrome and organ dysfunction, particularly renal and pulmonary dysfunction, in noncardiac surgical settings^40^. Higher MCP-1 levels correlated with increased inotropic support in children after cardiopulmonary bypass^27^ and with higher risk of acute kidney injury and death after cardiac operations in adults^41^. Additional studies support the role of MCP-1 as a key amplifier of inflammation, as increased MCP-1 levels are associated with worse clinical outcomes in trauma/hemorrhagic shock patients, including prolonged mechanical ventilation and extended stays in intensive care unit^42^. In vitro studies have shown that CCL11/Eotaxin increases endothelial cell permeability^43^.

The above studies indicate a clinical impact of cytokine exposure from RBC-primed CPB. Therefore, reducing cytokines through PBUF is necessary and beneficial, as demonstrated by the following studies. Two studies have already shown that PBUF could mitigate an intra- and postoperative rise in inflammatory markers such as Procalcitonin, TNF-α, IL-1β, IL-6 and CXCL8/IL-8 in children undergoing CPB heart surgery^44,45^. In these studies, PBUF was associated with reduced inotropic support, shorter ventilation times and intensive care stays. Our current institutional approach to PBUF focuses on normalizing pH, glucose and electrolytes. A single filtration cycle is effectively counteracting the ongoing mediator release from RBCs and reduces the mediator content, but falls short in reducing some cytokine concentrations below patient’s baseline. However, there are potential strategies to improve the efficacy of PBUF: increasing the number of filtration cycles, pre-treating RBCs to remove supernatants using blood cell processor^46^ or cell saver devices^47^ or incorporating techniques such as countercurrent dialysis^48^, might further reduce mediator levels. In addition to PBUF, different ultrafiltration methods during CPB surgery are an established strategy for reducing the cytokine load during cardiac surgery and improving postoperative outcomes. Numerous studies have been conducting applying various ultrafiltration methods, such as modified ultrafiltration, zero-balance ultrafiltration, and combination techniques, as well as different filter types^14,21^.

When evaluating the effectiveness of PBUF for RBC-primed CPB, several factors must be considered, including the selection of components for sanguinous priming, priming volumes, the PBUF process, and the CPB circuit setup. These factors can differ between centers and affect study findings and patient outcomes diversely. A comparison with a study by Bierer et al. highlights these differences^49^. Bierer et al. used a sanguineous priming solution containing both RBC and fresh frozen plasma (FFP), whereas our study used RBC exclusively. Unlike RBCs, FFP contains also significant amounts of complement factors^50^, which were the main focus of Bierer et al.’s study. Additionally, Bierer et al. used a different CPB system setup with a Terumo Capiox® Hemoconcentrator HCO5 containing a Polysulfone membrane for PBUF, whereas we applied a polyarylsulfone membrane in our study providing different filtration properties. Interestingly, Bierer et al. demonstrated a reduction in cytokine concentrations after CPB initiation, primarily due to extreme hemodilution. In their sanguine group, 500 mL of priming volume were applied to patients with a median weight of 4.9 (3.4–6.6) kg. Considering a blood volume of 70–80 mL per kg, this corresponds to a ratio of about 1.4–1.5:1 between the priming volume and the patient’s blood volume. In our study, we applied a priming volume of 240 ml in patients with a median (range) weight of 5.0 (3.1.−10.5) kg. Furthermore, they used a limited cytokine panel and included only one of the eight relevant mediators that we identified as exceeding the patients’ plasma concentrations. This highlights the importance of considering various components of CPB priming in assessing patient outcomes and the challenges of comparing results from different studies.

Our study faces several limitations. First, the results obtained from RBC supernatants and CPB prime were not corrected for potential residual leukocyte and platelet contamination. However, our RBCs were leukodepleted, which generally reduces white blood cells by almost 99.99% and total leukocyte content to fewer than 5 × 10⁶ per unit of blood. Second, the proportion of positive samples for each cytokine and sample type, as well as the median mediator concentration for the cytokine-positive samples are provided. This approach accounts for the large inter-individual variability and documents the likelihood of a specific mediator being present and its expected concentration if detected. Consequently, the overall median, encompassing both positive and negative samples, is generally lower than the median reported here for most mediators. Third, due to the overall low concentration for some cytokines in the prime, the removal effect of PBUF could only be assessed for mediators that were reliably detectable in the prime. Fourth, the low observation count is a limitation, despite focusing on a specific patient cohort. For ethical reasons, we collected patient samples after anesthesia induction to minimize the number of venous punctures. Therefore, it is possible that anesthetic drugs may have influenced patients’ baseline mediator values. Finally, we chose the median preoperative baseline concentration of patients for specific cytokines as an arbitrary threshold for the potential clinical relevance of mediator load in RBC priming. However, this approach does not fully exclude the possibility that lower concentrations or levels of mediators may have an effect on the immune system in children undergoing cardiac surgery.

In conclusion, multiple cytokines and chemokines were present in RBCs and were significantly released after priming the CPB circuit. PBUF effectively removed inflammatory mediators via the effluent, but with the current approach of a single filtration cycle, their concentrations remained largely stable. This limitation is likely due to the fact that the convective elimination of mediators through the hemofilter is combined with simultaneous volume reduction and the filtration characteristics of each cytokine. Beyond filtration, the decrease in total mediator content may be due to adsorption to circuit components or re-binding to RBCs. Improved washing techniques may further optimize mediator levels in RBC-primed CPB circuits.

## Methods

### Study design

This prospective observational study was performed at Hannover Medical School between October 2019 and January 2021. The study was approved by the local ethics committee of the Hannover Medical School (No. 8591_BO_S_2019). All procedures involving human participants were performed in accordance with the ethical standards of the institutional and national research committee and with the Helsinki Declaration of 1964 and its later amendments or comparable ethical standards. Written informed consent was obtained for each child from their legal guardians. Trial registration: DRKS, DRKS00027572. Registered 07 February 2025—Retrospectively registered, https://www.drks.de/DRKS00027572.

### Study population

Children with a body weight of less than 10 kg scheduled for congenital heart surgery with RBC priming of the CPB system were eligible for enrollment. Exclusion criteria were known immunodeficiency, suspected preoperative infection, and preoperative mechanical ventilation. Written informed consent was obtained for each child from their legal guardians.

### Cardiopulmonary bypass

General anesthesia was induced intravenously with etomidate, sufentanil, esketamine and atracurium and maintained at the discretion of the anesthesiologist. The CPB system (Stöckert S5, Munich, Germany) and setup (Terumo FX05 Oxygenator, Eschborn, Germany; Terumo 3/16"× 1/4"tubing set, Eschborn, Germany) were standardized for all operations of the 22 patients (Fig. 1). Heparin-coated tubing was used in all systems. CPB circuits were uniformly primed with 10 mL/kg of 20% human albumin (max. 100 mL), 1 mL of 10% calcium, 3 mL/kg of 20% mannitol, bicarbonate-buffered hemofiltration solution (Duosol, B. Braun, Melsungen, Germany), and heparin (150 IE/kg). After replacing the pre-bypass filter, 125 mL of CPB-buffered RBCs, stored in saline adenine-glucose-mannitol solution, was added to the circuit. Packed RBCs were used as provided by the blood bank without additional washing. Subsequently, circuit prime blood gas and electrolyte levels were analyzed to identify metabolic abnormalities. PBUF was performed at the perfusionists’ discretion using a hollow-fiber polyarylsulfone membrane hemofilter (Maquet BC 20 Plus, Rastatt, Germany)^17^, with a maximum pressure of 300 mmHg and a standard PBUF flow rate of approximately 0.1 L/min, until pH status and electrolyte levels were normalized. Before cannulation, circuit volume was further reduced to the CPB circuit priming volume of 240 mL through continued PBUF.

### Study protocol

Blood samples were collected from stored, unwashed RBC units, from the CPB prime 1 min after RBCs were added and distributed throughout the circuit, and from CPB prime following PBUF immediately before cannulation. Additional control samples were collected directly from asanguine primes from three of the CPB circuits before RBC addition. All prime circuit samples were collected from the venous line (Fig. 1). For each time point, 0.5–1.0 ml sample volume was collected in EDTA tubes (Sarstedt, Nümbrecht, Germany) and immediately cooled on ice until further processing. PBUF effluent samples were collected from the hemofilter outflow line (Fig. 1) at the end of PBUF process in uncoated sterile sample tubes, simultaneously with the second CPB prime sample. Baseline patient samples were collected after induction of anesthesia and central venous line placement before sternotomy. All samples were centrifuged (2000 rpm, 8 min, 4 °C), and supernatants were frozen at −80 °C for later analysis.

Mediator concentrations in plasma/PBUF effluent/RBC supernatant were quantified using Luminex-based multiplex technology and Bio-Plex assays (Bio-Plex Pro Human Cytokine Screening Panel, 48-Plex and Bio-Plex Pro Human Cytokine VCAM-1 and ICAM-1 Set, Fa Bio-Rad, Hercules, Ca, USA) as previously described according to manufacturer’s instructions^51^. All samples were diluted 1:1 with sample diluent provided with the kits. Standards were reconstituted and prepared according to the manufacturer’s instructions. All samples were measured in one experiment on 8 plates performed with one solution of beads, secondary antibody and SA-PE. One standard curve was used for all plates and concentrations were determined using the Bio-Plex Manager 6.1 software. Intra-assay recovery rates were determined for the standard curve as percentage of fit between calculated concentrations and the mathematical regression and these resulted in 95–105% fit. Six baseline samples were measured in a separate analysis and were not included in further analyses. Laboratory data, patient clinical data, and CPB-related data were documented throughout the entire study.

The following 50 mediators were analyzed: Cutaneous T Cell attracting chemokine (CTACK, CCL27), CCL11/Eotaxin, basic fibroblast growth factor (FGF-β), Granulocyte-colony stimulating factor (G-CSF), Granulocyte–macrophage colony-stimulating factor (GM-CSF), Growth-regulated alpha protein (GRO-α, CXCL1), Hepatocyte growth factor (HGF), Intracellular adhesion molecule 1 (ICAM-1), Interferon-gamma (IFN-g) a2, Interleukin (IL) 1a, 1b, 2, 3, 4, 5, 6, 7, 8, 9, 10, 12(p40), 12(p70), 13, 15, 16, 17, 18, Interleukin receptor antagonist 1 (IL-1RA), soluble IL-2 receptor alpha (IL-2Rα), Interferon-gamma-inducible protein 10 (IP-10, CXCL10), Leukemia inhibitory factor (LIF), Monocyte chemotactic protein (MCP) 1 (CCL2) and 3 (CCL7), Macrophage colony-stimulating factor (M-CSF), Macrophage migration inhibitory factor (MIF), Monokine induced by interferon gamma (MIG, CXCL9), Macrophage inflammatory protein (MIP) 1α (CCL3) and 1β (CCL4), Nerve growth factor β (β-NGF), Platelet-derived growth factor-BB (PDGF-bb), Regulated upon activation normal T cell expressed and presumably secreted (RANTES, CCL5), Stem cell factor (SCF), Stem cell growth factor β (SCGFβ), Stromal cell-derived factor 1a (SDF-1α, CXCL12), Tumor necrosis factor (TNF) α and β, Tumor necrosis factor-related apoptosis-inducing ligand (TRAIL), Vascular cell adhesion molecule 1 (VCAM-1), and vascular endothelial growth factor (VEGF).

### Statistical analysis

Statistical analysis was performed in R (version 2022.07.2). All analyte concentration data showed non-normal distributions and are presented as median and interquartile range. Median concentrations were calculated from all samples that were above the lower limit of quantification. To enable comparative statistical analyses including mediator-negative samples, the lowest measurable concentration for each respective cytokine was used in statistical hypothesis testing for mediator-negative samples. The correlation between cytokine concentration and RBC storage time was evaluated using the Kendall´s rank correlation. Comparisons of concentrations between two time points were performed using the Wilcoxon Rank-Sum test (RBC vs. CPB prime pre-PBUF vs. patient baseline, RBC vs. CPB prime after PBUF vs. patient baseline) or the Wilcoxon Signed-Rank Test (CPB pre-PBUF vs. after PBUF) from the stats package. A p-value of < 0.05 was considered statistically significant. The removal effect^15,52^ was calculated as follows:

$$Removal Effect =\frac{1}{n} \sum_{i=1}^{n}\left(\frac{Cytokine Concentration in Ultrafiltration {Effluent}_{i}}{Cytokine Concentration in CPB {Prime}_{i}}\right)$$

For the calculation of cytokine removal, only those sample pairs were included in which the concentration in the prime was at least twice the lower limit of quantification. The absolute cytokine load was estimated from the CPB volume and the measured concentration during prime preparation before and after PBUF.

## Supplementary Information

Supplementary Information.

## Data Availability

The datasets used and/or analyzed during the current study are available from the corresponding author on reasonable request.

## Abbreviations

CPB: Cardiopulmonary bypass
CTACK: Cutaneous T-Cell attracting chemokine
FGF: Basic fibroblast growth factor
GM-CSF: Granulocyte–macrophage colony-stimulating factor
G-CSF: Granulocyte colony-stimulating factor
GRO: Growth-regulated alpha protein
ICAM: Intracellular adhesion molecule
IL: Interleukin
IP-10: Interferon-gamma-inducible protein
MCP: Monocyte chemotactic protein
M-CSF: Macrophage colony-stimulating factor
MIF: Macrophage migration inhibitory factor
MIP: Macrophage inflammatory protein
NGF: Nerve growth factor
PBUF: Pre-bypass ultrafiltration
RANTES: Regulated upon activation normal T cell expressed and presumably secreted
RBC: Red blood cell
SCF: Stem cell factor
SCGF: Stem cell growth factor
TNF: Tumor necrosis factor
TRAIL: Tumor necrosis factor-related apoptosis-inducing ligand
VCAM: Vascular cell adhesion molecule
VEGF: Vascular endothelial growth factor
