# Supplementary material for: Pre-bypass ultrafiltration reduces cytokine burden of blood prime in pediatric cardiac surgery
Source: Sci Rep. 2025 Aug 25;15:31271. doi: 10.1038/s41598-025-15746-7 (PMC12379243; doi:10.1038/s41598-025-15746-7)
Supplement: Supplementary file 1 — Supplementary Information. [file 41598_2025_15746_MOESM1_ESM.pdf]

Pre-Bypass Ultrafiltration reduces Cytokine Burden of Blood Prime in Pediatric Cardiac Surgery

Supplementary Information

Pages

Supplementary Results

RBC supernatants and storage duration 2

Supplementary Discussion

RBC supernatants and storage duration 2

Supplementary Figures

Figure S1: Correlation of cytokine concentration and storage time 3

Figure S2: Longitudinal course of cytokine concentrations during prime preparation .... 4 - 8

Figure S3: Cytokine concentrations in pre-bypass ultrafiltration effluent for all investigated cytokines ... 9 - 13

Figure S4: Longitudinal course of total cytokine load during prime preparation 14

Supplementary Tables

Table S1: Patient demographics 15

Table S2: Concentration of cytokines in asanguineous priming .... 16 - 17

Table S3: Filtration of cytokines .... 18 - 20

Table S4: Absolute load of all cytokines in RBC, priming solution before & after PBUF .... 21 - 24

Supplementary Results

RBC supernatants and storage duration

The median duration of RBC storage before use was 12.5 days (range 7–28 days). Five of 50 cytokines, namely Stem cell growth factor b (SCGFb, CCL11/Eotaxin, Interferon-gamma-inducible protein 10 (CXCL10/IP-10), (Supplementary Figure S1), IL-16 and Cutaneous T-Cell attracting chemokine (CCL27/CTACK), (data not shown) showed a positive correlation between cytokine concentrations in RBC supernatants and storage duration.

Supplementary Discussion

Remarkably, of the 50 mediators analyzed, only five showed a correlation between mediator burden and prolonged storage time. This is in contrast to the literature, which describes changes in immunological profiles of leukocyte-depleted RBCs depending on storage time or manufacturing methods [1,2]. A possible explanation for these discrepancies is that our study did not track mediator concentrations in packed RBCs across the complete storage period. The absence of repetitive analyses of RBC supernatants during storage represent a limitation of our study. Our results on mediator accumulation only reflect a correlation between storage time and cytokine content. These effects could also be attributed to inter-individual donor-specific differences in packed RBCs.

1 Bal, S. H. *et al.* Effect of storage period of red blood cell suspensions on helper T-cell subpopulations. *Blood Transfus* **16**, 262-272 (2018). https://doi.org:10.2450/2017.0238-16

2 Almizraq, R. J. *et al.* Blood manufacturing methods affect red blood cell product characteristics and immunomodulatory activity. *Blood Adv* **2**, 2296-2306 (2018). https://doi.org:10.1182/bloodadvances.2018021931

Supplementary Figure S1: Correlation of cytokine concentration and storage time

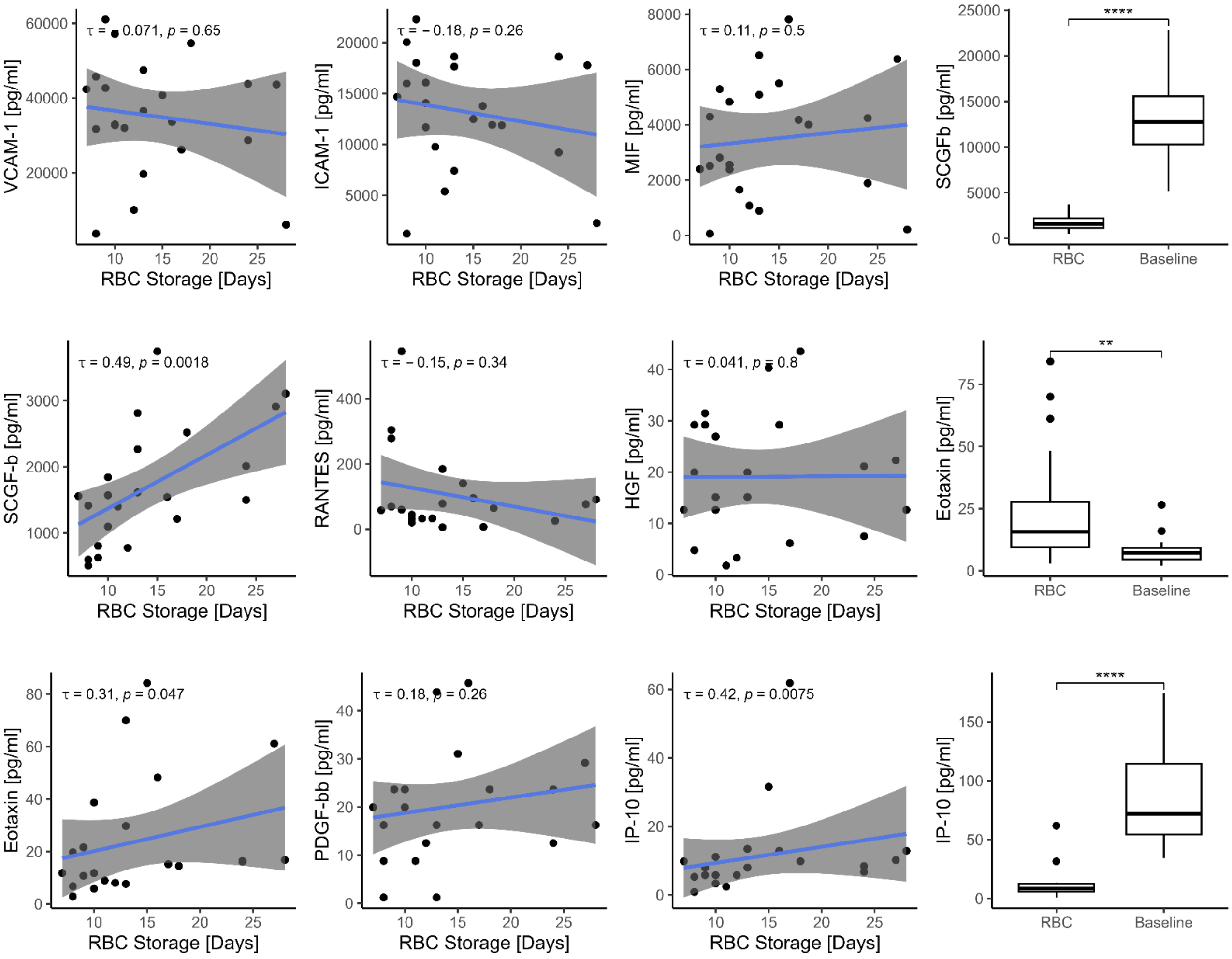

Figure S1. Examples of the levels of the 9 most frequently detected cytokines as a function of storage time. Of the 50 measured cytokines only SCGFβ, CCL11/Eotaxin and CXCL10/IP-10 (d,g,i) as well as IL-16 and CCL27/CTACK (data not shown) showed a positive correlation between storage time and cytokine load (Kendall rank correlation, $p<0.05$). j, k, l, Median levels of SCGFβ, CXCL10/IP-10 and CCL11/Eotaxin in RBC supernatants compared to the preoperative baseline concentrations of patients (Wilcoxon rank-sum test; $p < 0.01$; $p < 0.001$).

Supplementary Figure S2: Longitudinal course of cytokine concentrations during prime preparation

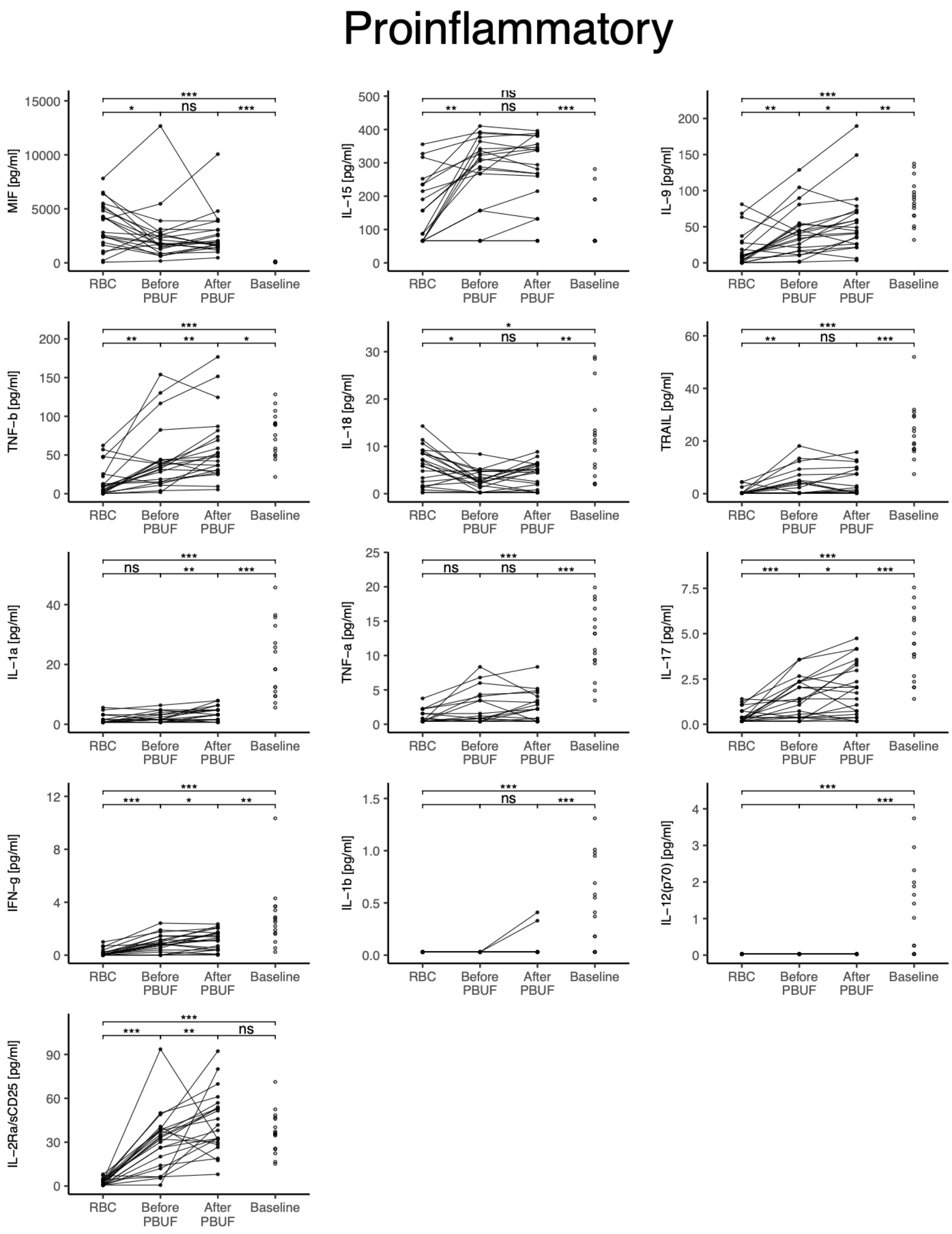

Antiinflammatory

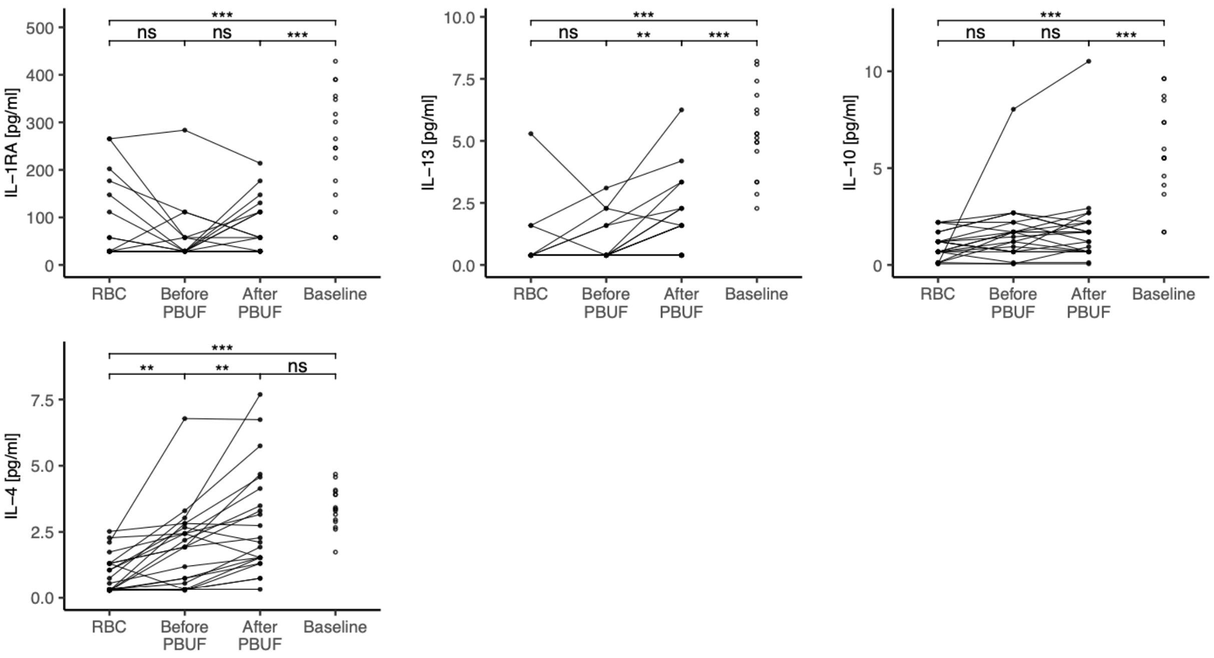

Multifunctional

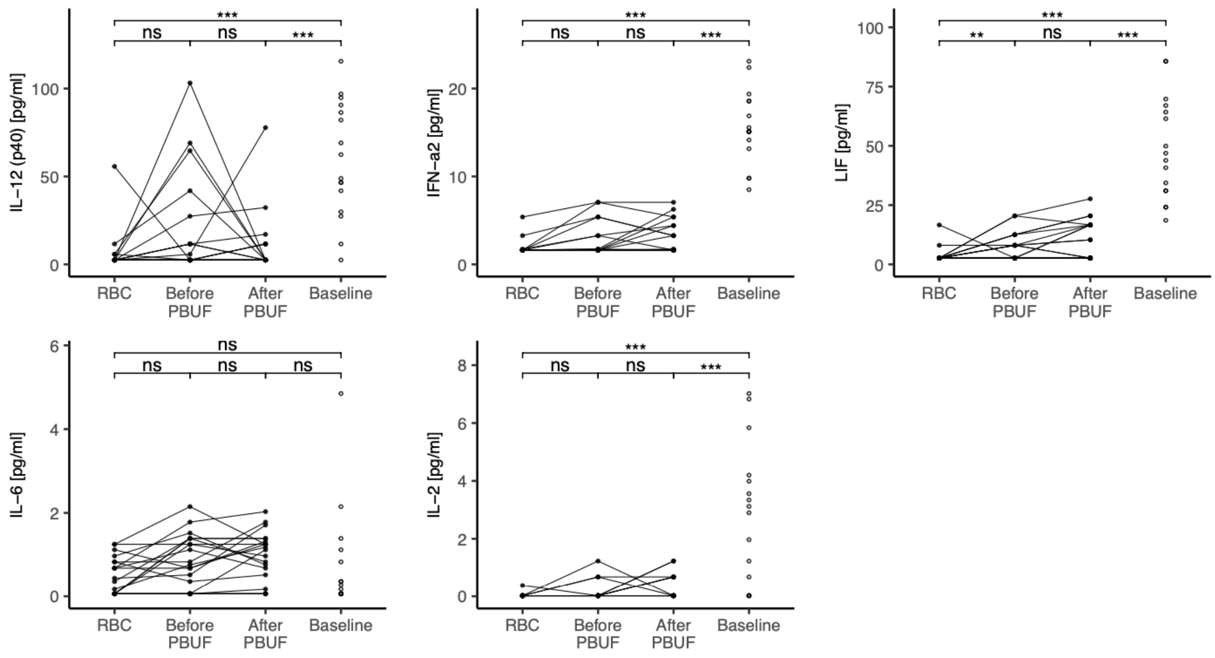

Chemokines

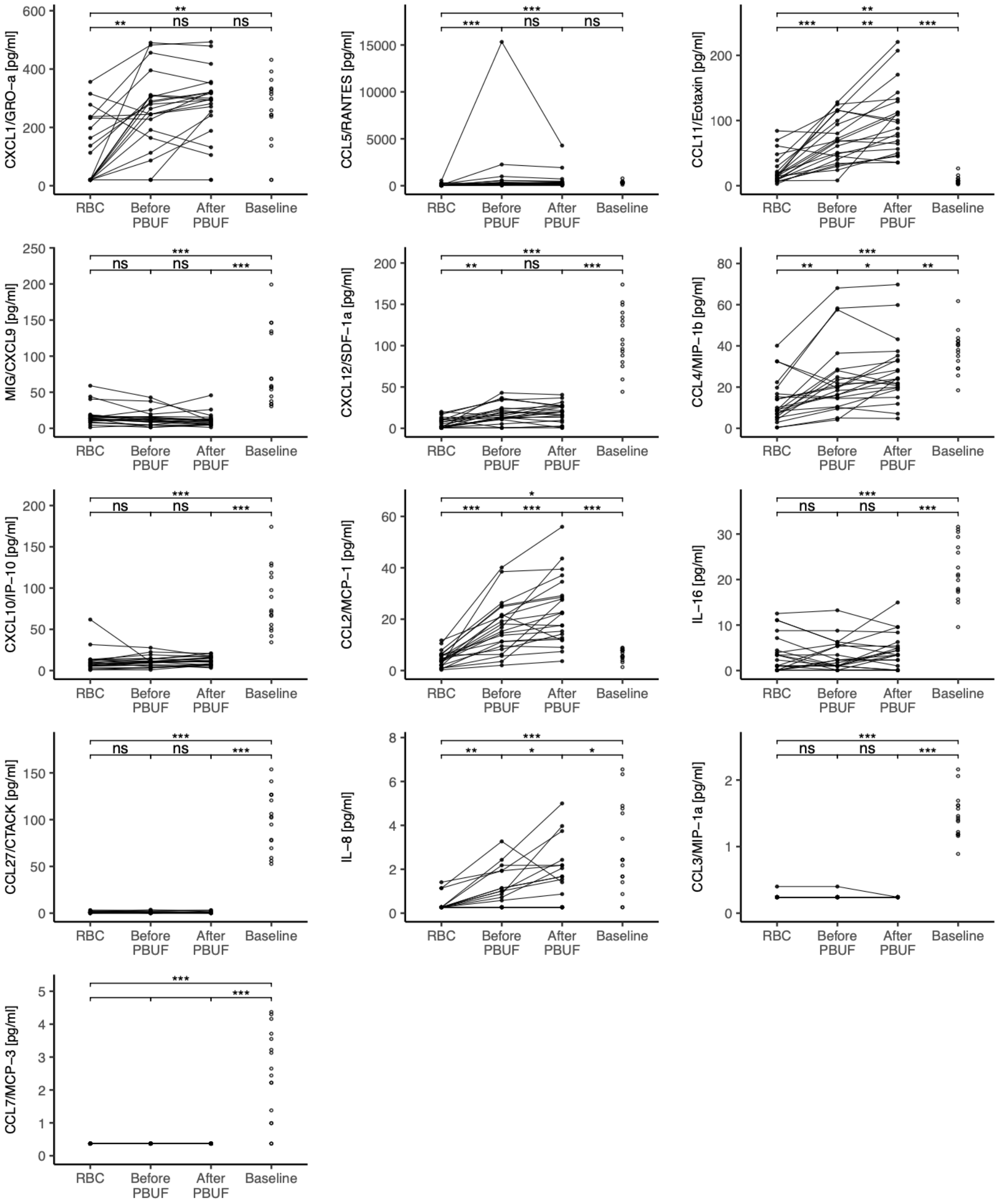
CXCL1/GRO−a [pg/ml]
CCL5/RANTES [pg/ml]
CCL11/Eotaxin [pg/ml]
MIG/CXCL9 [pg/ml]
CXCL12/SDF−1a [pg/ml]
CCL4/MIP−1b [pg/ml]
CXCL10/IP−10 [pg/ml]
CCL2/MCP−1 [pg/ml]
IL−16 [pg/ml]
CCL27/CTACK [pg/ml]
IL−8 [pg/ml]
CCL3/MIP−1a [pg/ml]
CCL7/MCP−3 [pg/ml]
RBC
Before PBUF
After PBUF
Baseline

Growth_Factors

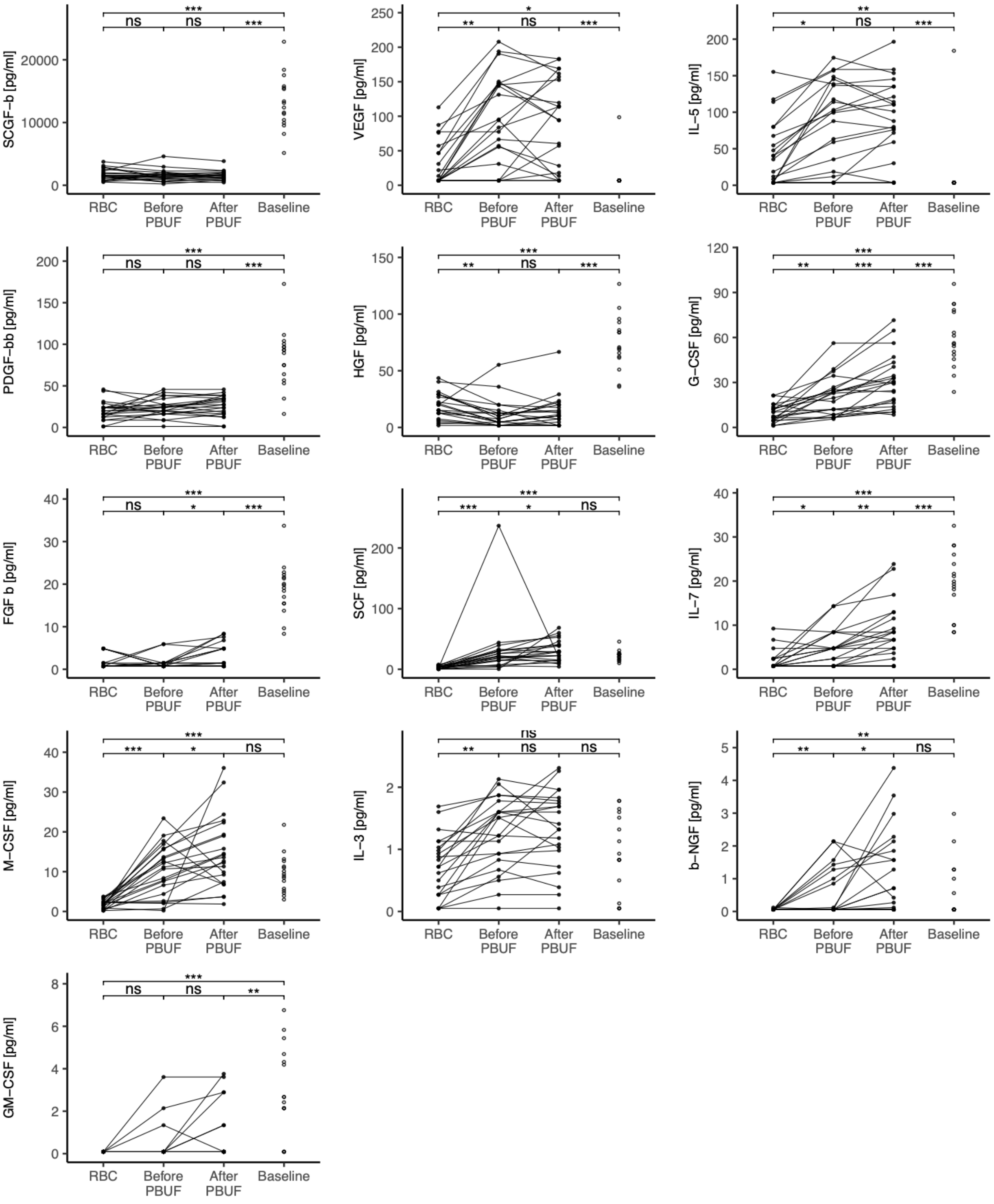

Endothelial_Markers

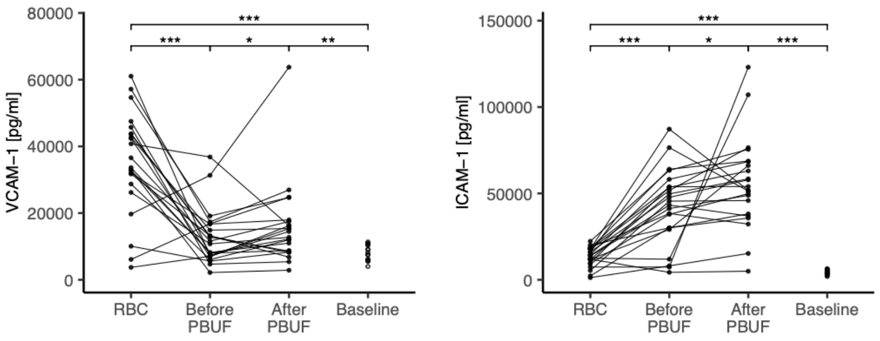

Figure S2. Longitudinal course of 42 out of 50 cytokines during prime preparation. Figures show mediator concentrations in red blood cell (RBC) supernatants and prime samples before pre-bypass ultrafiltration (PBUF) and after PBUF. *p<0.05, **p<0.01, ***p<0.001.

Supplementary Figure S3: Cytokine concentrations in pre-bypass ultrafiltration effluent for all investigated cytokines

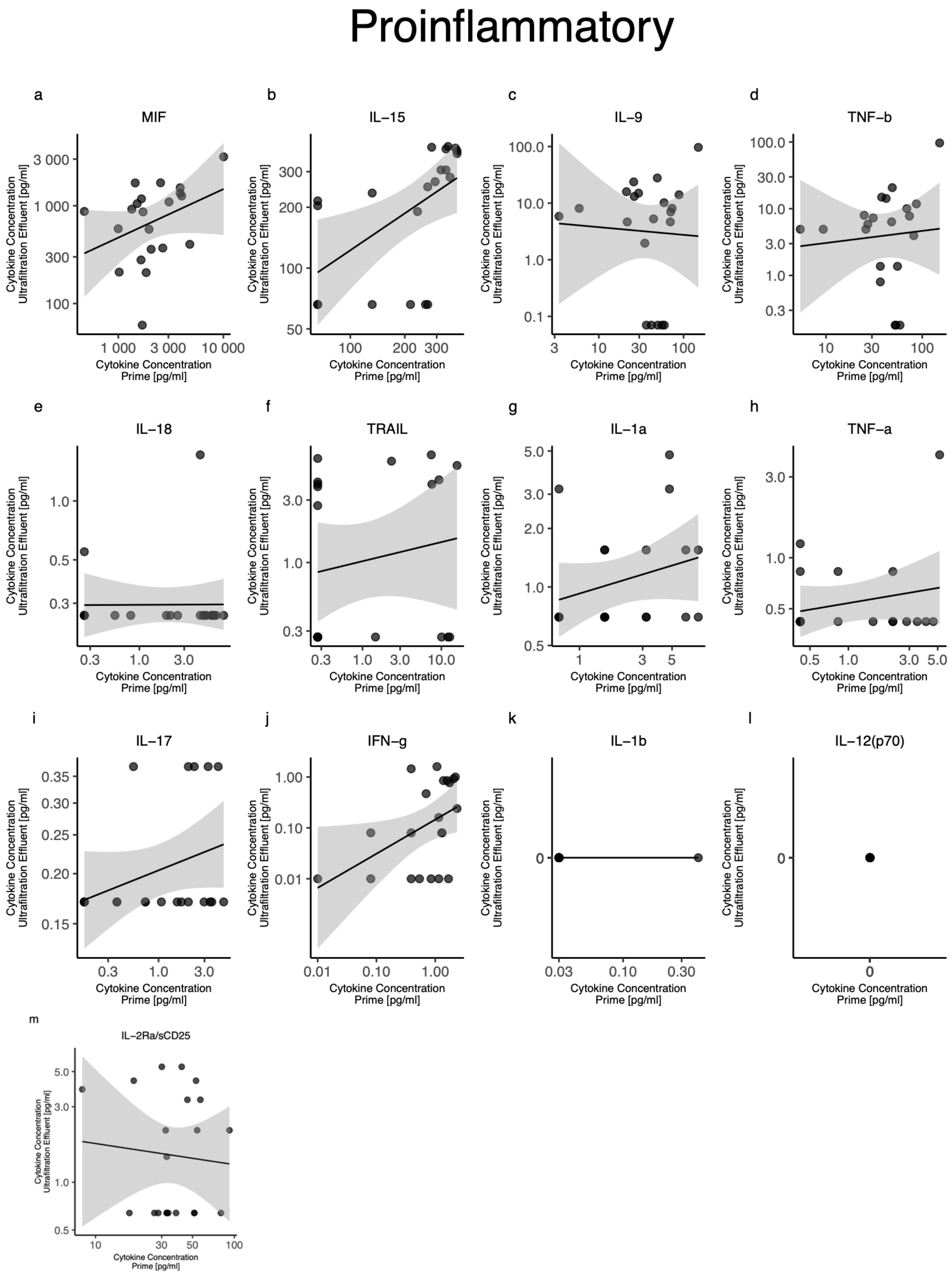

Antiinflammatory

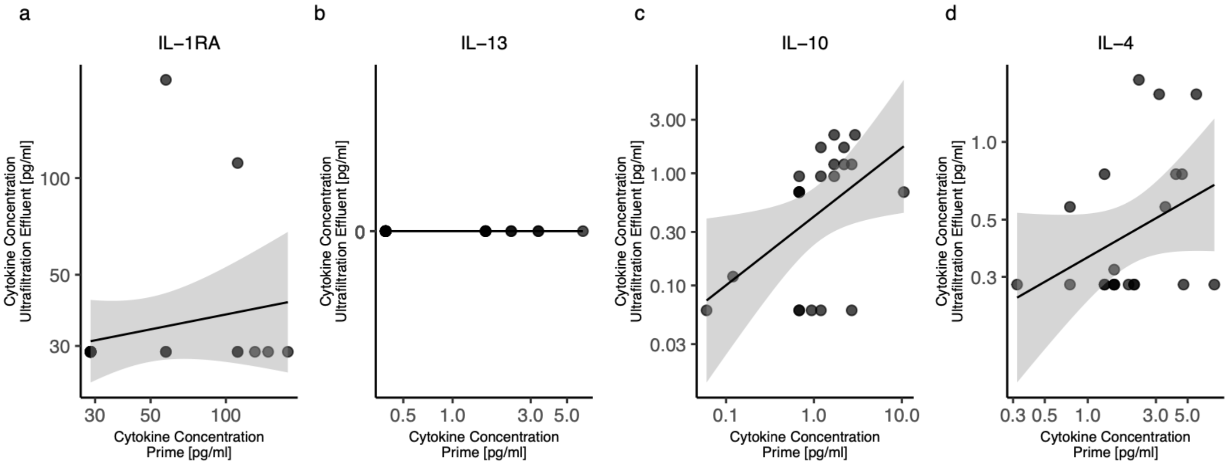

Multifunctional

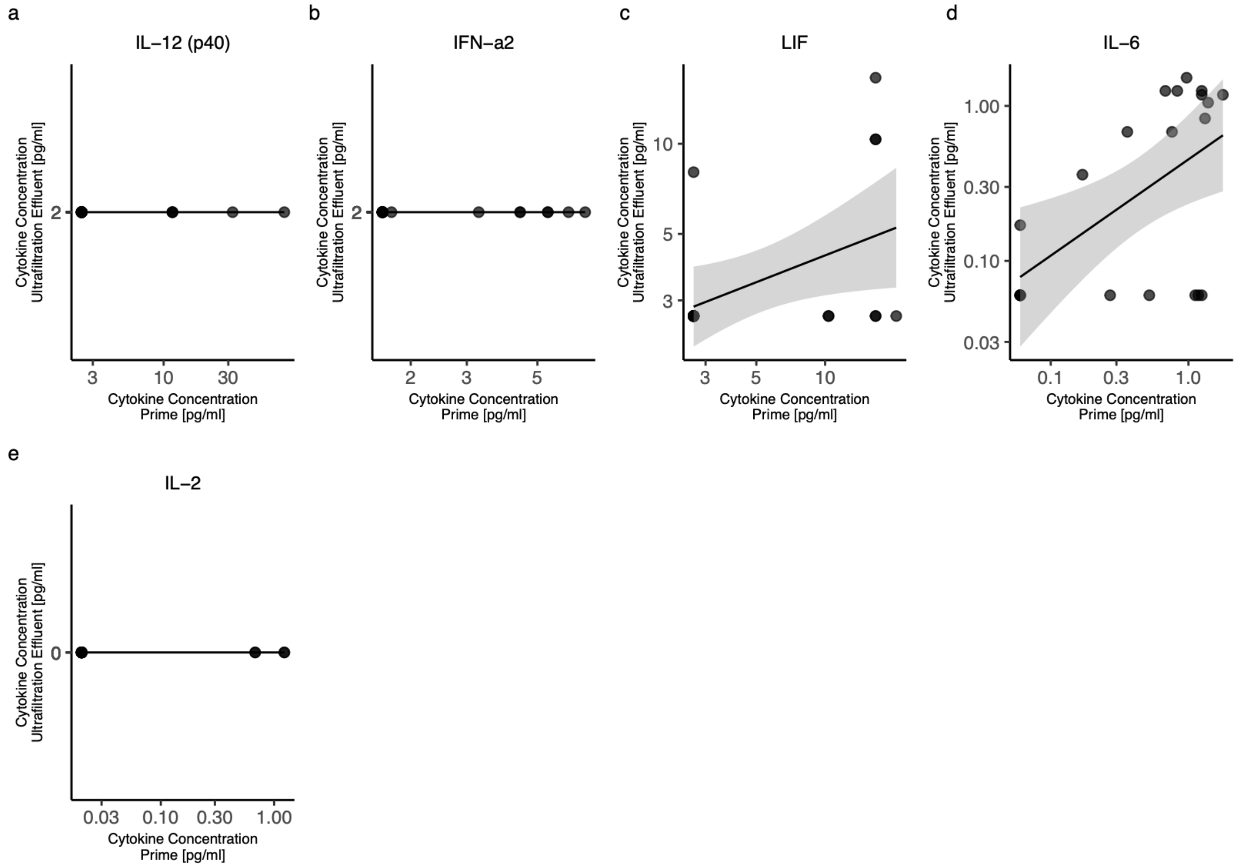

Chemokines

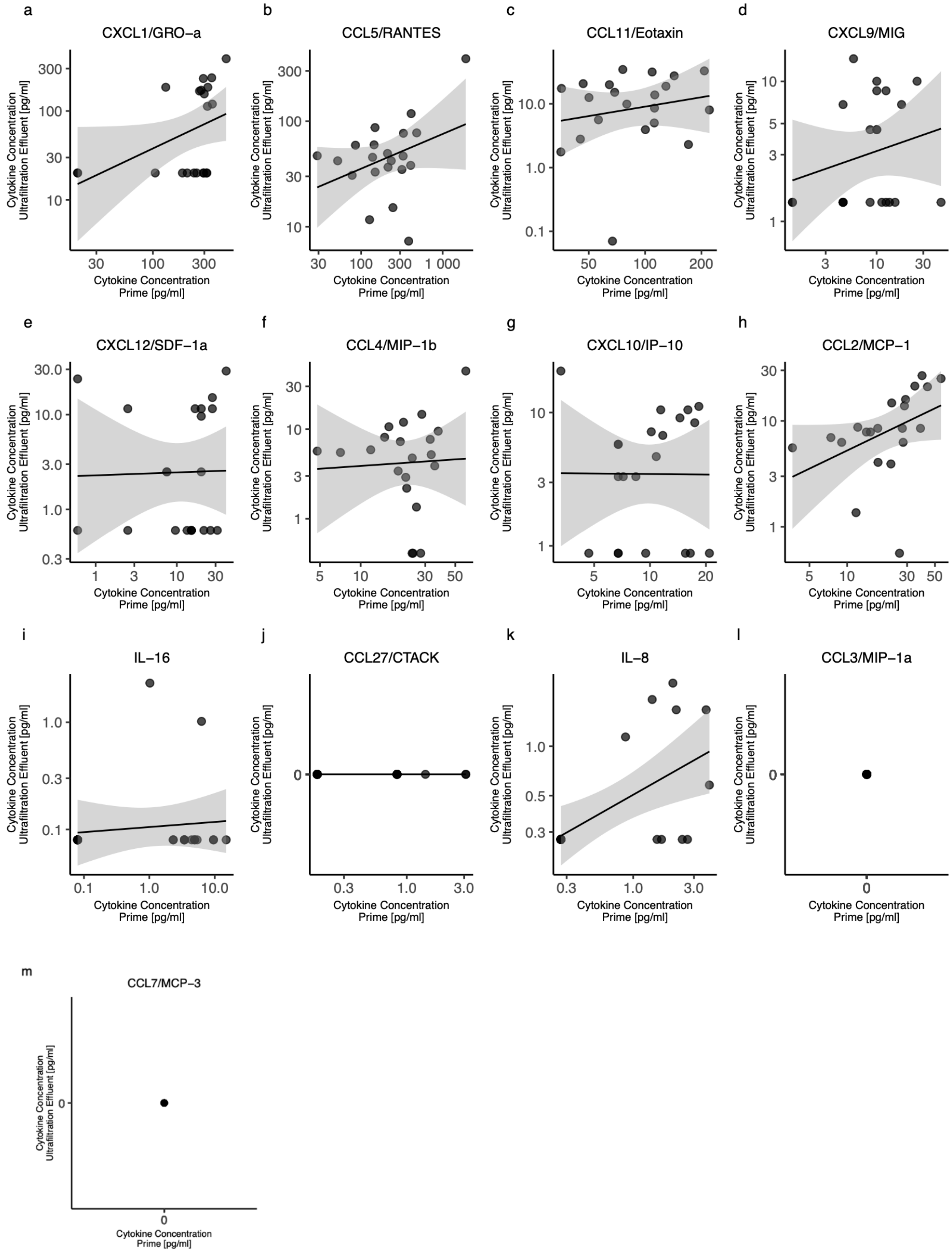
a
CXCL1/GRO−a
b
CCL5/RANTES
c
CCL11/Eotaxin
d
CXCL9/MIG
e
CXCL12/SDF−1a
f
CCL4/MIP−1b
g
CXCL10/IP−10
h
CCL2/MCP−1
i
IL−16
j
CCL27/CTACK
k
IL−8
l
CCL3/MIP−1a
m
CCL7/MCP−3
Cytokine Concentration
Ultrafiltration Effluent [pg/ml]
Cytokine Concentration
Prime [pg/ml]

Growth_Factors

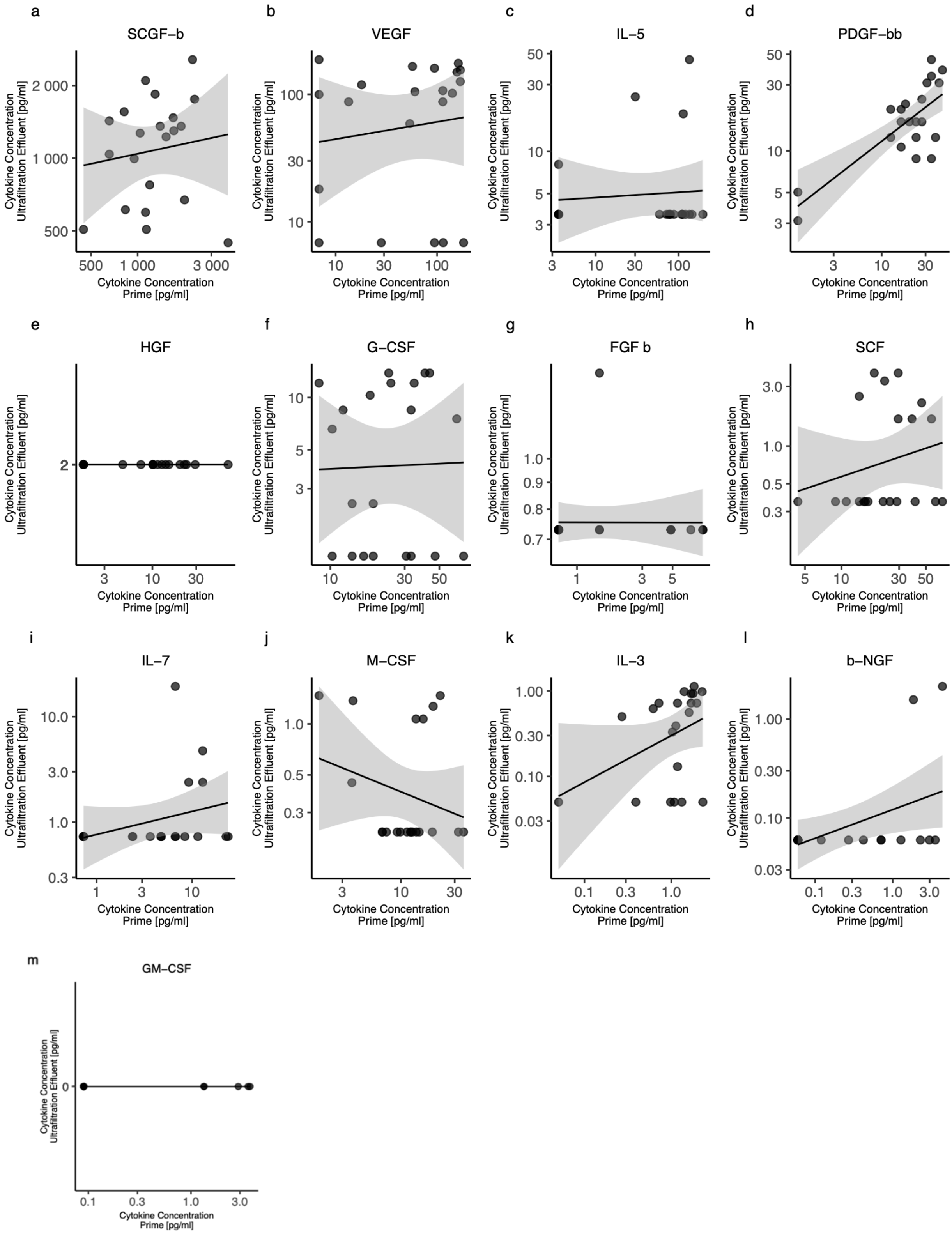

Endothelial_Markers

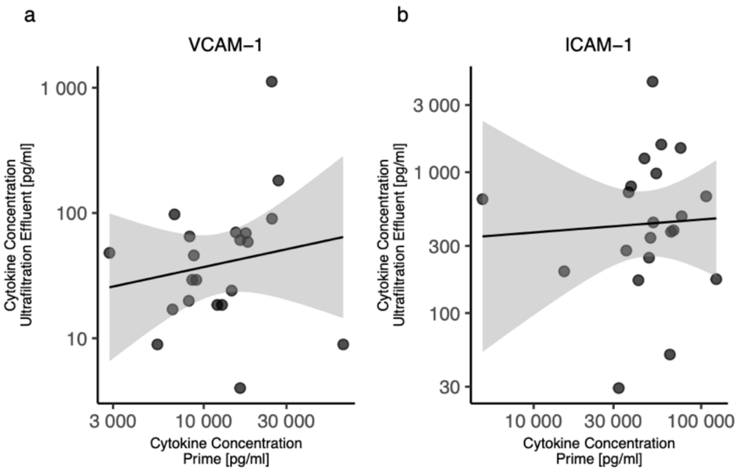

Figure S3. The figure illustrates the spot concentrations of cytokines in the pre-bypass ultrafiltration (PBUF) effluent during the terminal phase of PBUF in relation to the concomitant mediator concentrations of CPB priming for all investigated cytokines.

Supplementary Figure S4: Longitudinal course of total cytokine load during prime preparation

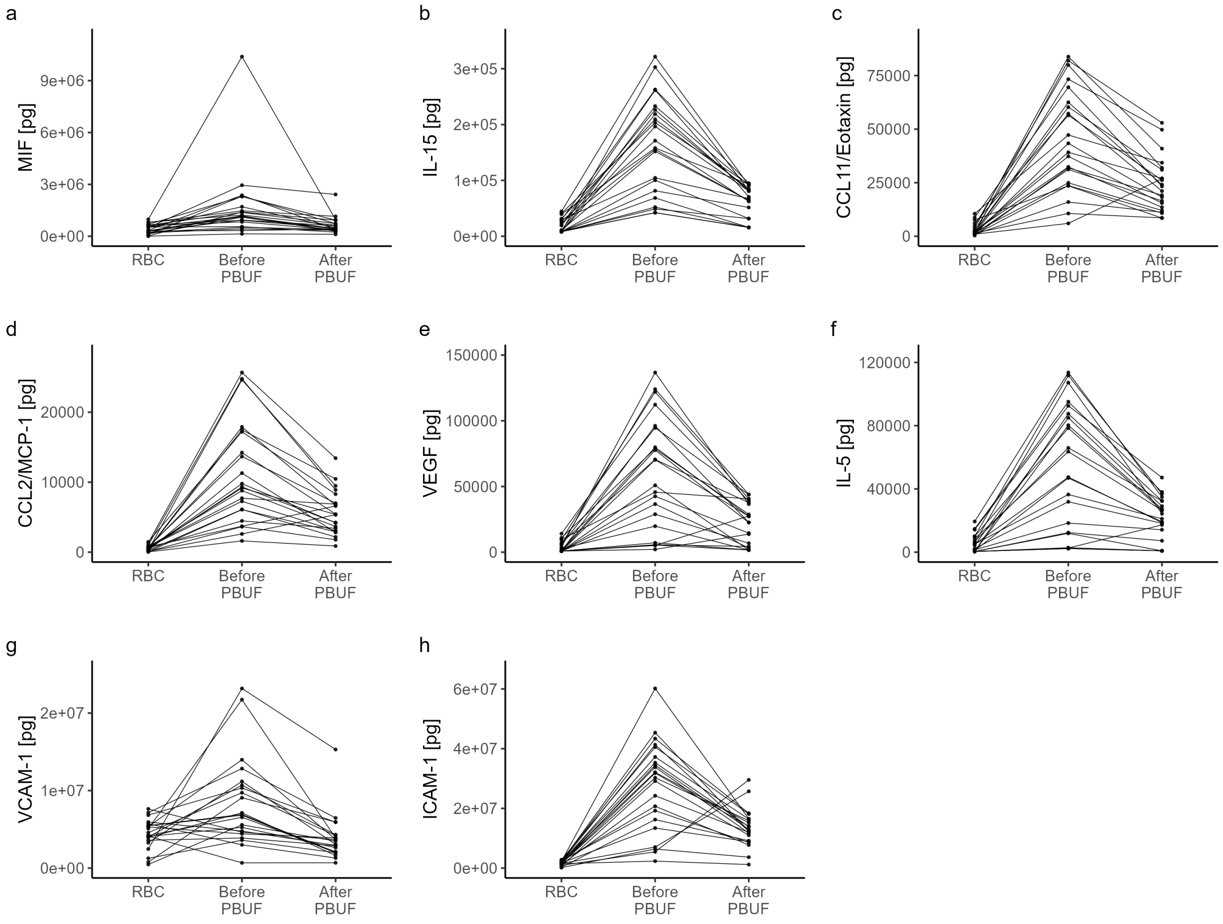

Figure S4. a-h) Total mediator load in [pg] of exemplary cytokines during prime preparation. The mediator concentration was multiplied by the volume of the applied packed red blood cells (RBCs) and the respective filling volume of the CPB circuit at certain stages of the priming process, taking into account the particular volume used for pre-bypass ultrafiltration (PBUF). Figure shows the total mediator load in applied RBCs, before and after PBUF of the eight cytokines (MIF, IL-15, CCL11/Eotaxin, CCL2/MCP-1, VEGF, IL-5, VCAM-1 and ICAM-1) exceeding the median patient baseline concentration. ICAM, Intracellular adhesion molecule; IL, Interleukin; MCP, Monocyte chemotactic protein, MIF, Macrophage migration inhibitory factor; VCAM, Vascular cell adhesion molecule; VEGF, Vascular endothelial growth factor.

Supplementary Table S1: Patient demographics

Patient number	Sex [f= female, m= male]	Age at surgery [years, months,	Weight at surgery [kg]	Congenital heart defect	Surgical procedure
1	m	0 y 1 m 6 d	4.1	CoA, AoV stenosis	Aortic arch reconstruction, aortic valve commissurotomy
2	m	0 y 2 m 27 d	4.6	ASD II, perimembranous VSD, PDA	ASD II + VSD closure, PDA ligation
3	f	0 y 2 m 13 d	5.1	DORV, AoV atresia, hypoplastic aortic arch, CoA, ASD II; Bilateral PAB	Damus–Kaye–Stansel procedure, Sano shunt, PA debanding
4	f	0 y 2 m 18 d	3.6	Multiple VSDs	VSD closure
5	f	0 y 10 m 27 d	7.1	TOF, severe valv. + supravalv. PS, ASD II	TOF correction with VSD + ASD II closure, RVOT myectomy, transannular patch repair
6	f	1 y 10 m 21 d	9.6	PA-VSD, LPA stenosis; RVOT reconstruction	VSD closure, RV-PA conduit
7	f	0 y 3 m 28 d	5.6	Complex univentricular heart (single inlet) with ventricle inversion, TA, RV hypoplasia, VSD, malposition of great arteries, PS, ASD II	Damus-Kay-Stansel procedure, BT-Shunt, PA reconstruction, atrial septectomy, PDA ligation
8	m	0 y 5 m 26 d	4.9	Perimembranous VSD	VSD closure
9	m	1 y 5 m 13 d	7.0	PA-IVS, severe TV hypoplasia; BT-Shunt, LPA reconstruction, atrial septectomy	PCPC + LPA reconstruction
10	m	1 y 8 m 8 d	10.5	DORV, MV dysplasia; PAB	VSD closure, PA reconstruction, ASD enlargement
11	f	0 y 6 m 14 d	5.1	AVSD, LAVV regurgitation	AVSD repair
12	m	0 y 0 m 10 d	3.5	d-TGA, VSD, ASD II	ASO, ASD II + VSD closure
13	f	0 y 0 m 14 d	4.0	Simple d-TGA, ASD II	ASO, ASD II closure
14	m	0 y 0 m 16 d	3.0	Simple d-TGA, ASD II	ASO, ASD II closure
15	f	0 y 4 m 7 d	4.7	Multiple VSDs, ASD II	ASD II + VSD closure
16	m	0 y 1 m 5 d	3.1	d-TGA, muscular VSD; Ballon atrioseptostomy	ASO, ASD II + VSD closure
17	m	0 y 5 m 6 d	5.5	Malalignment type VSD, right aortic arch	VSD closure, ligamentum arteriosum transection
18	f	0 y 4 m 21 d	5.1	VSD, PDA, TV dysplasia	VSD closure, PDA ligation, TV reconstruction
19	m	0 y 4 m 28 d	5.1	Perimembranous VSD	VSD closure
20	m	0 y 5 m 16 d	5.8	Partial AVSD	ASD I closure, AVV reconstruction
21	f	0 y 0 m 10 d	3.2	Simple d-TGA	ASO, ASD II closure
22	m	0 y 0 m 16 d	3.3	d-TGA, VSD; Ballon atrioseptostomy	ASO, ASD II + VSD closure

Table S1. Overview of patient demographics including sex, age, weight, congenital heart defect and surgical procedure. AoV, Aortic valve; ASD, Atrial septal defect; ASO, Arterial switch operation; AVSD, Atrioventricular septal defect; AVV, Atrioventricular valve; BT-Shunt, Blalock-Taussig shunt; CoA, Coarctation of the aorta; DORV, Double outlet right ventricle; d-TGA, Dextro-transposition of the great arteries; LAAV, Left atrioventricular valve; LPA, Left pulmonary artery; MV, Mitral valve; PA, Pulmonary artery; PAB, Pulmonary artery banding; PA-IVS, Pulmonary atresia with intact ventricular septum; PA-VSD, Pulmonary atresia with ventricular septal defect; PCPC, Partial Cavo-Pulmonary Connection; PDA, Patent ductus arteriosus; PS, Pulmonary stenosis; RV, Right ventricle; RVOT, Right ventricular outflow tract; TA, Tricuspid atresia; TOF, Tetralogy of Fallot; TV, Tricuspid valve; VSD, Ventricular septal defect.

Supplementary Table S2: Concentration of cytokines in asanguineous priming

	Cytokine	Asanguineous Priming 1 [pg/ml]	Asanguineous Priming 2 [pg/ml]	Asanguineous Priming 3 [pg/ml]
proinflammatory	MIF	0	0	0
	IL-15	0	0	0
	IL-9	0	0	0
	TNF-β	0	0	0
	IL-18	0	0	0
	TRAIL	0	0.92	0
	IL-1α	0	0	0
	TNF-α	0	0	0
	IL-17	0	0	0
	IFN-γ	0	0	0
	IL-1β	0	0	0
	IL-12(p70)	0	0	0
	IL-2Rα	17.91	31.09	24.21
anti-inflammatory	IL-1RA	0	0	0
	IL-13	0	0	0
	IL-10	0	0	0
	IL-4	0	0	0
multifunctional	IL-12 (p40)	0	0	0
	IFN-α2	0	0	0
	LIF	0	0	0
	IL-6	0	0	0
	IL-2	0	0	0
chemokines	GRO-α/CXCL1	0	0	0
	RANTES/CCL5	0	0	0
	Eotaxin/CCL11	0	0	0
	MIG/CXCL9	0	0	0
	SDF-1α	0	0	0
	MIP-1β/CCL4	0	0	0
	IP-10/CXCL10	0	0	0
	MCP-1/CCL2	0	0	0
	IL-16	0	0	0
	CTACK/CCL27	0	0	0
	IL-8/CXCL8	0	0	0

	MIP-1α/CCL3	0	0	0
	MCP-3/CCL7	0	0	0
growth factors	SCGF-β	1844.51	4022.63	1627.11
	VEGF	0	0	0
	IL-5	0	0	0
	PDGF-bb	0	0	0
	HGF	0	0	0
	G-CSF	0	0	0
	FGF-β	0	0	0
	SCF	0	5.39	5.39
	IL-7	0	0	0
	M-CSF	8.35	10.15	8.10
	IL-3	0	0	0
	β-NGF	0	0	0
	GM-CSF	0	0	0
endothelial markers	VCAM-1	0	0	0
	ICAM-1	0	0	0

Table S2. Concentration [pg/ml] of the 50 measured cytokines in asanguineous priming solutions (n=3). CCL, C-C-Motif Chemokine Ligand; CTACK, Cutaneous T-Cell attracting chemokine (CCL27); CXCL, CXC-Motif Chemokine Ligand; FGF-β, Basic fibroblast growth factor; GM-CSF, Granulocyte-macrophage colony-stimulating factor; GRO-a, Growth-regulated alpha protein (CXCL1); HGF, Hepatocyte growth factor; ICAM, Intracellular adhesion molecule; IFN-γ, Interferon-gamma; IL, Interleukin; IL-1RA, Interleukin receptor antagonist 1; IL-2Rα, soluble IL-2 receptor alpha, IP-10, Interferon-gamma-inducible protein 10 (CXCL10); LIF, Leukemia inhibitory factor; MCP, Monocyte chemotactic protein; M-CSF, Macrophage colony-stimulating factor; MIF, Macrophage migration inhibitory factor; MIG, Monokine induced by interferon gamma; MIP, Macrophage inflammatory protein; NGF, Nerve growth factor; PDGF-bb, Platelet-derived growth factor-BB; RANTES, Regulated upon activation normal T cell expressed and presumably secreted (CCL5); SCF, Stem cell factor; SCGF, Stem cell growth factor; SDF-1α, Stromal cell-derived factor 1α; TNF, Tumor necrosis factor; TRAIL, Tumor necrosis factor-related apoptosis-inducing ligand; VCAM, Vascular cell adhesion molecule; VEGF, Vascular endothelial growth factor.

Supplementary Table S3: Filtration of cytokines

Cytokine	Priming solution after PBUF		PBUF effluent		Removal effect
	**Median** (IQR) [pg/ml]	(Out of 22)	**Median** (IQR) [pg/ml]	(Out of 22)	(SD)
**MIF**	**1902.82** (1517.94)	(22)	**923.18** (811.77)	(19)	**50.41%** (42.50%)
**IL-15**	**337.26** (106.91)	(18)	**305.89** (136.21)	(13)	**85.73%** (52.65%)
**IL-9**	**46.60** (41.96)	(22)	**8.09** (9.43)	(15)	**32.04%** (39.16%)
**TNF-β**	**48.44** (42.66)	(22)	**6.82** (5.56)	(16)	**23.29%** (24.61%)
**IL-18**	**5.07** (3.18)	(18)	**0.41** (0.58)	(4)	**4.14%** (11.10%)
**TRAIL**	**7.52** (8.49)	(12)	**4.20** (1.87)	(10)	**n.a.**
**IL-1α**	**3.99** (2.39)	(16)	**1.55** (1.64)	(8)	**n.a.**
**TNF-α**	**3.16** (2.39)	(13)	**0.83** (0.38)	(5)	**n.a.**
**IL-17**	**2.04** (2.45)	(19)	**0.37** (0)	(6)	**n.a.**
**IFN-γ**	**1.30** (1.14)	(21)	**0.62** (0.80)	(14)	**n.a.**
**IL-1β**	**0.37** (0.04)	(2)	**n.a.**	(0)	**n.a.**
**IL-12(p70)**	**n.a.**	(0)	**n.a.**	(0)	**n.a.**
**IL-1RA**	**111.40** (81.52)	(11)	**156.84** (45.44)	(2)	**n.a.**
**IL-2Rα**	**39.90** (23.19)	(22)	**3.32** (2.25)	(11)	**7.40%** (11.86%)
**IL-13**	**1.94** (1.49)	(14)	**0.39** (0)	(1)	**n.a.**
**IL-10**	**1.70** (1.52)	(21)	**1.07** (0.90)	(14)	**n.a.**
**IL-4**	**2.20** (2.45)	(22)	**0.75** (0.97)	(9)	**19.71%** (26.60%)
**IL-12 (p40)**	**11.61** (13.09)	(7)	n.a.	(0)	**n.a.**
**IFN-α2**	**4.41** (2.12)	(10)	n.a.	(0)	**n.a.**

**LIF**	**16.62** (12.04)	(14)	**9.20** (6.35)	(6)	**n.a.**
**IL-6**	**1.25** (0.56)	(17)	**1.18** (0.45)	(11)	**72.35%** (55.34%)
**IL-2**	**0.68** (0.28)	(7)	n.a.	(0)	**n.a.**
**GRO-α/CXCL1**	**298.23** (63.36)	(20)	**178.26** (61.59)	(10)	**39.34%** (40.82%)
**RANTES/CCL5**	**224.01** (256.78)	(22)	**45.46** (23.72)	(19)	**34.06%** (36.91%)
**Eotaxin/CCL11**	**92.49** (66.75)	(22)	**13.82** (15.06)	(19)	**18.69%** (15.06%)
**MIG/CXCL9**	**10.02** (6.67)	(21)	**8.57** (3.19)	(9)	**46.14%** (67.35%)
**SDF-1α**	**20.00** (12.71)	(20)	**11.53** (4.13)	(10)	**25.88%** (28.55%)
**MIP-1β/CCL4**	**24.01** (13.35)	(22)	**5.79** (3.91)	(16)	**34.64%** (31.87%)
**IP-10/CXCL10**	**11.57** (9.14)	(22)	**6.96** (5.19)	(14)	**72.88%** (136.03%)
**MCP-1/CCL2**	**22.38** (15.95)	(22)	**8.5** (9.08)	(19)	**54.66%** (31.58%)
**IL-16**	**4.44** (2.85)	(17)	**1.67** (0.65)	(2)	**1.48%** (4.90%)
**CTACK/CCL27**	**0.83** (0.65)	(19)	**0.18** (0)	(5)	**n.a.**
**IL-8/CXCL8**	**1.86** (0.93)	(14)	**1.67** (0.59)	(6)	**n.a.**
**MIP-1α/CCL3**	**0.24** (0)	(1)	**n.a.**	(0)	**n.a.**
**MCP-3/CCL3**	**1464.06** (737.51)	(22)	**n.a.**	(0)	**n.a.**
**SCGFβ**	**114.34** (91.74)	(18)	**1298.95** (872.34)	(19)	**97.41%** (54.74%)
**VEGF**	**110.43** (56.66)	(18)	**122.28** (57.94)	(14)	**97.73%** (83.92%)
**IL-5**	**27.35** (16.62)	(21)	**18.59** (16.50)	(5)	**8.97%** (22.11%)
**PDGF-bb**	**12.01** (12.47)	(20)	**16.25** (15.76)	(19)	**85.23%** (36.33%)
**HGF**	**29.54** (22.17)	(22)	**1.77** (0)	(3)	**n.a.**
**G-CSF**	**1902.82** (1517.94)	(22)	**12.08** (4.48)	(11)	**32.25%** (38.23%)
**FGFβ**	**4.86** (5.54)	(12)	**1.46** (0)	(1)	**n.a.**
**SCF**	**28.35** (25.07)	(22)	**2.22** (1.67)	(9)	**4.73%** (6.87%)

**IL-7**	**8.42** (6.26)	(17)	**2.39** (2.36)	(5)	**36.99%** (89.54%)
**M-CSF**	**13.12** (11.37)	(22)	**1.07** (1.06)	(10)	**8.44%** (19.13%)
**IL-3**	**1.41** (0.75)	(21)	**0.72** (0.31)	(13)	**43.08%** (32.38%)
**β-NGF**	**1.57** (1.54)	(14)	**1.86** (0.29)	(2)	**n.a.**
**GM-CSF**	**0.37** (0)	(2)	**n.a**.	(0)	**n.a.**
**VCAM-1**	**2.89** (1.70)	(6)	**53.41** (48.79)	(18)	**0.67%** (1.03%)
**ICAM-1**	**13614.31** (8532.63)	(22)	**486.50** (576.71)	(19)	**2.19%** (3.22%)

Table S3. Concentration in [pg/ml] of all of the 50 cytokines analyzed in the priming solution after pre-bypass ultrafiltration (PBUF) and in the PBUF effluent. The removal effect indicates the elimination capacity by filtration. n.a., Not applicable.

CCL, Chemokine C-C-Motif ligand; CTACK, Cutaneous T-Cell attracting chemokine (CCL27); CXCL, CXC-Motif chemokine ligand; FGF-β, Basic fibroblast growth factor; G-CSF, Granulocyte-Colony Stimulating Factor; GM-CSF, Granulocyte-macrophage colony-stimulating factor; GRO-α, Growth-regulated alpha protein (CXCL1); HGF, Hepatocyte growth factor; ICAM, Intracellular adhesion molecule; IFN-γ, Interferon-gamma; IL, Interleukin; IL-1RA. Interleukin receptor antagonist 1; IL-2Rα, soluble IL-2 receptor alpha; IP-10, Interferon-gamma-inducible protein 10 (CXCL10); IQR, Interquartile range; LIF, Leukemia inhibitory factor; MCP, Monocyte chemotactic protein; M-CSF, Macrophage colony-stimulating factor; MIF, Macrophage migration inhibitory factor; MIG, Monokine induced by interferon gamma; MIP, Macrophage inflammatory protein; NGF, Nerve growth factor; PBUF, Pre-bypass ultrafiltration; PDGF-bb, Platelet-derived growth factor-BB; RANTES, Regulated upon activation normal T cell expressed and presumably secreted (CCL5); SCF, Stem cell factor; SCGF, Stem cell growth factor; SD, standard deviation; SDF-1α, Stromal cell-derived factor 1α; TNF, Tumor necrosis factor; TRAIL, Tumour necrosis factor-related apoptosis-inducing ligand; VCAM, Vascular cell adhesion molecule; VEGF, Vascular endothelial growth factor.

Supplementary Table S4: Absolute load of all cytokines in RBC, priming solution before and after PBUF

	Cytokine	RBC		Priming solution before PBUF		Priming solution after PBUF		% relative to pre-PBUF value
		**Median**<br>(IQR)<br>[pg]	(Out of 22)	**Median**<br>(IQR)<br>[pg]	(Out of 22)	**Median**<br>(IQR)<br>[pg]	(Out of 22)	(SD)
**pro-inflammatory**	MIF	**426361**<br>(376452)	(22)	**1166526** ^{yyy}<br>(792100)	(22)	**456677** ***<br>(364304)	(22)	**52.4%**<br>(26.7%)
	IL-15	**28125**<br>(13958)	(12)	**203429** ^{yyy}<br>(77644)	(17)	**80942** ***<br>(25657)	(18)	**41.6%**<br>(11.3%)
	IL-9	**1334**<br>(2774)	(18)	**23664** ^{yyy}<br>(21780)	(22)	**11184** ***<br>(10070)	(22)	**52.6%**<br>(20.1%)
	TNF-b	**1196**<br>(2471)	(19)	**23080** ^{yyy}<br>(15367)	(22)	**11624** ***<br>(10239)	(22)	**54.1%**<br>(20.2%)
	IL-18	**839**<br>(695)	(20)	**2147** ^{yy}<br>(1358)	(18)	**1217** **<br>(763)	(18)	**61.8%**<br>(31.5%)
	TRAIL	**555**<br>(119)	(3)	**3046** ^{yyy}<br>(4486)	(13)	**1804** **<br>(2037)	(12)	**n.a.**
	IL-1a	**399**<br>(304)	(7)	**1302** ^{yyy}<br>(1163)	(13)	**956** **<br>(574)	(16)	**n.a.**
	TNF-a	**195**<br>(175)	(9)	**2214** ^{yyy}<br>(1816)	(13)	**758** **<br>(574)	(13)	**n.a.**
	IL-17	**91**<br>(88)	(11)	**1125** ^{yyy}<br>(1098)	(20)	**490** ***<br>(587)	(19)	**n.a.**
	IFN-g	**20**<br>(53)	(11)	**589** ^{yyy}<br>(506)	(18)	**312** ***<br>(274)	(21)	**n.a.**
	IL-1b	**n.a.**	(0)	**19** ^{yyy}<br>(0)	(1)	**89** **<br>(10)	(2)	**n.a.**
	IL-12(p70)	**n.a.**	(0)	**n.a.** $^{n.a.}$	(0)	**n.a.** $^{n.a.}$	(0)	**n.a.**
**anti-inflammatory**	IL-1RA	**20274**<br>(15014)	(8)	**48494** ^{yyy}<br>(28337)	(6)	**26736** **<br>(19565)	(11)	**n.a.**
	IL-2Ra	**481**<br>(178)	(14)	**20250** ^{yyy}<br>(16948)	(21)	**9575** **<br>(5565)	(22)	**65.6%**<br>(52.47%)
	IL-13	**199**<br>(150)	(5)	**367** ^{yyy}<br>(943)	(11)	**464** $^{n.s.}$<br>(356)	(14)	**n.a.**
	IL-10	**150**<br>(81)	(20)	**1088** ^{yyy}<br>(901)	(20)	**408** ***<br>(365)	(21)	**n.a.**
	IL-4	**148**<br>(115)	(16)	**1402** ^{yyy}<br>(1212)	(21)	**527** ***<br>(587)	(22)	**58.1%**<br>(21.72%)

multifunctional	IL-12 (p40)	**718** (734)	(5)	**17523** (29050)	yyy	(9)	**2786** (3140)	*	(7)	**n.a.**
	IFN-a2	**409** (228)	(3)	**2663** (1771)	yyy	(8)	**1058** (509)	***	(10)	**n.a.**
	LIF	**333** (0)	(9)	**5146** (4787)	yyy	(14)	**3989** (2889)	***	(14)	**n.a.**
	IL-6	**85** (66)	(13)	**751** (537)	yyy	(15)	**300** (134)	***	(17)	**48.4%** (21.6%)
	IL-2	**25** (23)	(2)	**212** (415)	yyy	(6)	**163** (66)	n.s.	(7)	**n.a.**
chemokines	GRO-a/CXCL1	**29030** (11908)	(10)	**178880** (81039)	yyy	(19)	**71574** (15206)	***	(20)	**45.5%** (17.6%)
	RANTES/CCL5	**7801** (7805)	(22)	**111647** (167023)	yyy	(22)	**53762** (61626)	***	(22)	**46.3%** (34.5%)
	Eotaxin/CCL11	**1956** (2290)	(22)	**41276** (35464)	yyy	(22)	**22196** (16020)	***	(22)	**51.8%** (13.6%)
	MIG	**1814** (786)	(21)	**8343** (5525)	yyy	(20)	**2405** (1601)	***	(21)	**33.3%** (18.3%)
	SDF-1a	**1200** (1074)	(15)	**11632** (6681)	yyy	(19)	**4800** (3050)	***	(20)	**44.9%** (15.6%)
	MIP-1b/CCL4	**1190** (1304)	(20)	**12585** (8009)	yyy	(22)	**5761** (3205)	***	(22)	**51.3%** (33.4%)
	IP-10/CXCL10	**1049** (888)	(21)	**7117** (4758)	yyy	(21)	**2777** (2193)	***	(22)	**45.5%** (18.3%)
	MCP-1/CCL2	**540** (460)	(21)	**9252** (10372)	yyy	(22)	**5371** (3827)	***	(22)	**61.5%** (43.6%)
	IL-16	**429** (765)	(17)	**1546** (2675)	yyy	(20)	**1066** (684)	**	(17)	**69.1%** (51.5%)
	CTACK/CCL27	**104** (228)	(16)	**242** (752)	yy	(16)	**199** (156)	*	(19)	**n.a.**
	IL-8/CXCL8	**88** (109)	(6)	**730** (862)	yyy	(13)	**446** (222)	***	(14)	**n.a.**
	MIP-1a/CCL3	**30**	(8)	**236**	yyy	(1)	**58**	***	(1)	**n.a.**

growth factors	SCGF-b	**193746** (134628)	(22)	**959863** (568572)	yyy	(22)	**351374** (177003)	***	(22)	**43.9%** (23.2%)
	VEGF	**6486** (5282)	(10)	**77668** (50458)	yyy	(17)	**27440** (22018)	***	(18)	**34.4%** (18.4%)
	IL-5	**6392** (5410)	(14)	**72211** (52236)	yyy	(18)	**26503** (13597)	***	(18)	**43.4%** (13.0%)
	PDGF-bb	**2495** (926)	(21)	**14770** (11840)	yyy	(21)	**6564** (3989)	***	(21)	**42.9%** (10.9%)
	HGF	**2192** (2000)	(22)	**6826** (6659)	yy	(19)	**2882** (2993)	***	(20)	**n.a.**
	G-CSF	**1288** (885)	(20)	**13214** (12618)	yyy	(22)	**7088** (5320)	***	(22)	**56.6%** (17.8%)
	FGF b	**608** (425)	(7)	**934** (988)	yyy	(8)	**1166** (1330)	n.s.	(12)	**n.a.**
	SCF	**415** (403)	(18)	**13675** (10899)	yyy	(21)	**6803** (6016)	**	(22)	**61.9%** (68.9%)
	IL-7	**299** (416)	(7)	**3943** (2647)	yyy	(14)	**2021** (1502)	**	(17)	**65.0%** (24.4%)
	M-CSF	**256** (130)	(20)	**6840** (5676)	yyy	(22)	**3148** (2729)	**	(22)	**64.3%** (64.4%)
	IL-3	**97** (87)	(20)	**864** (522)	yyy	(21)	**338** (180)	***	(21)	**42.3%** (14.8%)
	b-NGF	**15** (0)	(1)	**786** (242)	yyy	(8)	**377** (368)	n.s.	(14)	**n.a.**
	GM-CSF	**n.a.**	(0)	**1379** (793)	yyy	(3)	**694** (409)	n.s.	(6)	**n.a.**
endothelial markers	VCAM-1	**4387664** (1785390)	(22)	**6866561** (5942788)	yy	(22)	**3267434** (2047830)	***	(22)	**51.1%** (22.0%)
	ICAM-1	**1739410** (937350)	(22)	**31044544** (19755528)	yyy	(22)	**12692652** (6658937)	**	(22)	**58.9%** (69.2%)

Table S4. Absolute mediator load [pg] of the 50 cytokines in utilized red blood cells (RBC), priming solution before and after pre-bypass ultrafiltration (PBUF), and the final mediator load in percent relative to pre-processing baseline levels before PBUF. To estimate the total cytokine load, the cytokine concentration was multiplied by the volume of the applied RBC and 2.) the respective filling volume of the CPB circuit at certain stages of the priming process.

Median concentrations were calculated from all samples that were above the lower limit of quantification. To enable comparative statistical analyses including mediator-negative samples, the lowest measurable concentration for each respective cytokine was used in statistical hypothesis testing for mediator-negative samples. Absolute cytokine load in utilized RBC versus priming solution before PBUF, y $p < 0.05$; yy $p < 0.01$; yyy $p < 0.001$; Cytokine load in priming solution

before PBUF versus after PBUF, * p <0.05; ** p <0.01; *** p <0.001. Median and Interquartile range (IQR) and number of cytokine-positive samples above detection limit are shown for each cytokine.

CCL, C-C-Motif Chemokine Ligand; CTACK, Cutaneous T-Cell attracting chemokine (CCL27); CXCL, CXC-Motif Chemokine Ligand; FGF-β, Basic fibroblast growth factor; G-CSF, Granulocyte-Colony Stimulating Factor; GM-CSF, Granulocyte-macrophage colony-stimulating factor; GRO-α, Growth-regulated alpha protein (CXCL1); HGF, Hepatocyte growth factor; ICAM, Intracellular adhesion molecule; IFN-γ, Interferon-gamma; IL, Interleukin; IL-1RA, Interleukin receptor antagonist 1; IL-2Rα, soluble IL-2 receptor alpha; IP-10, Interferon-gamma-inducible protein 10 (CXCL10); LIF, Leukemia inhibitory factor; MCP, Monocyte chemotactic protein; M-CSF, Macrophage colony-stimulating factor; MIF, Macrophage migration inhibitory factor; MIG, Monokine induced by interferon gamma; MIP, Macrophage inflammatory protein; NGF, Nerve growth factor; N.s., Not significant; PDGF-bb, Platelet-derived growth factor-BB; RANTES, Regulated upon activation normal T cell expressed and presumably secreted (CCL5); SCF, Stem cell factor; SCGF, Stem cell growth factor; SD, Standard deviation; SDF-1α, Stromal cell-derived factor 1α; TNF, Tumor necrosis factor; TRAIL, Tumor necrosis factor-related apoptosis-inducing ligand; VCAM, Vascular cell adhesion molecule; VEGF, Vascular endothelial growth factor.
